# Supplementary material for: A Humanized Yeast Phenomic Model of Deoxycytidine Kinase to Predict Genetic Buffering of Nucleoside Analog Cytotoxicity
Source: Genes (Basel). 2019 Sep 30;10(10):770. doi: 10.3390/genes10100770 (PMC6826991; doi:10.3390/genes10100770)
Supplement: Supplementary file 1 [file genes-10-00770-s001.zip › Additional_Files/Additional_File7_GOTermHeatmaps.zip!/Additional_File7_GOTermHeatmaps/regulation_of_protein_complex_assembly.pdf]

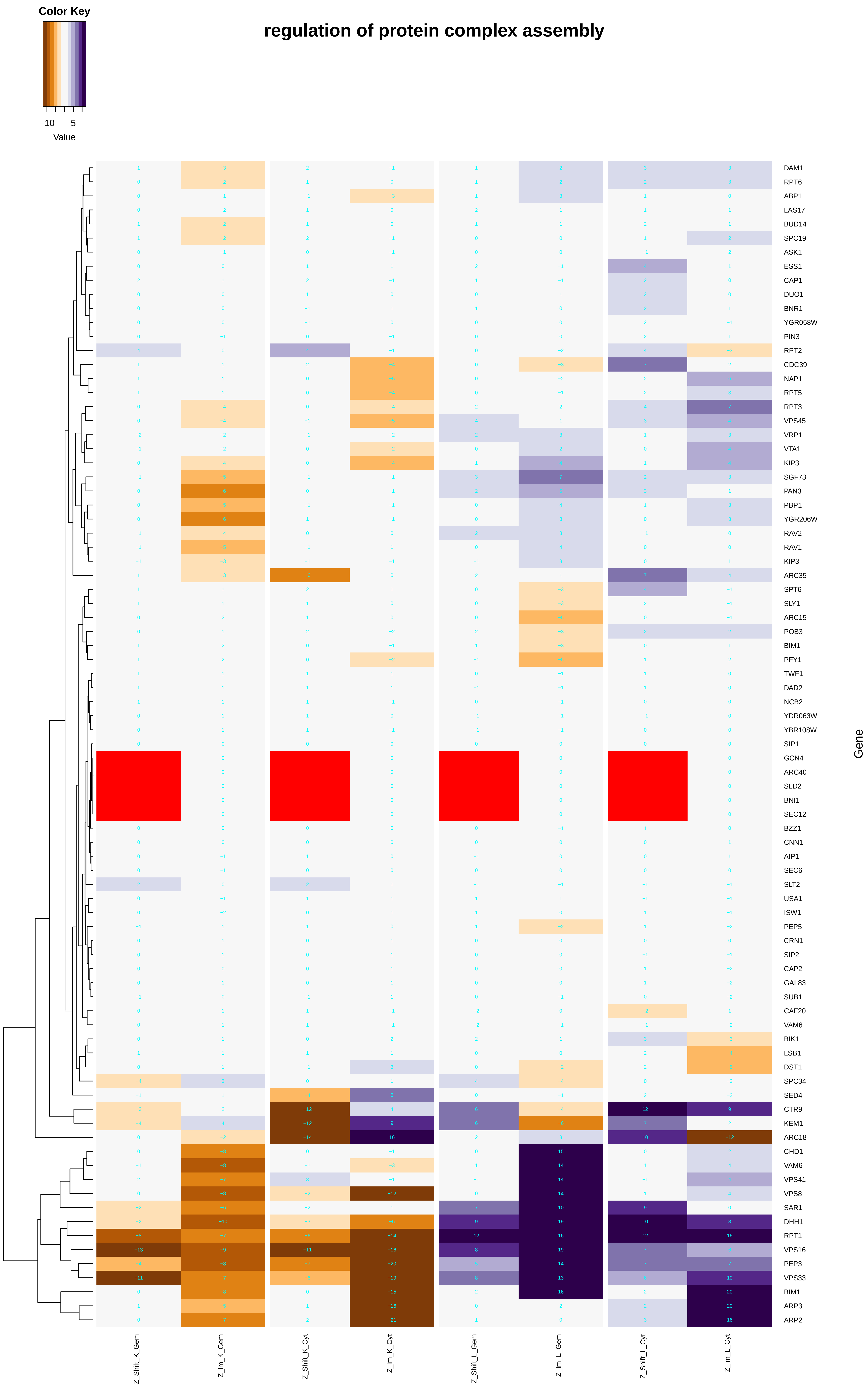
Color Key
−10
5
Value
regulation of protein complex assembly
DAM1
RPT6
ABP1
LAS17
BUD14
SPC19
ASK1
ESS1
CAP1
DUO1
BNR1
YGR058W
PIN3
RPT2
CDC39
NAP1
RPT5
RPT3
VPS45
VRP1
VTA1
KIP3
SGF73
PAN3
PBP1
YGR206W
RAV2
RAV1
KIP3
ARC35
SPT6
SLY1
ARC15
POB3
BIM1
PFY1
TWF1
DAD2
NCB2
YDR063W
YBR108W
SIP1
GCN4
ARC40
SLD2
BNI1
SEC12
BZZ1
CNN1
AIP1
SEC6
SLT2
USA1
ISW1
PEP5
CRN1
SIP2
CAP2
GAL83
SUB1
CAF20
VAM6
BIK1
LSB1
DST1
SPC34
SED4
CTR9
KEM1
ARC18
CHD1
VAM6
VPS41
VPS8
SAR1
DHH1
RPT1
VPS16
PEP3
VPS33
BIM1
ARP3
ARP2
Gene
Z_Shift_K_Gem
Z_lm_K_Gem
Z_Shift_K_Cyt
Z_lm_K_Cyt
Z_Shift_L_Gem
Z_lm_L_Gem
Z_Shift_L_Cyt
Z_lm_L_Cyt

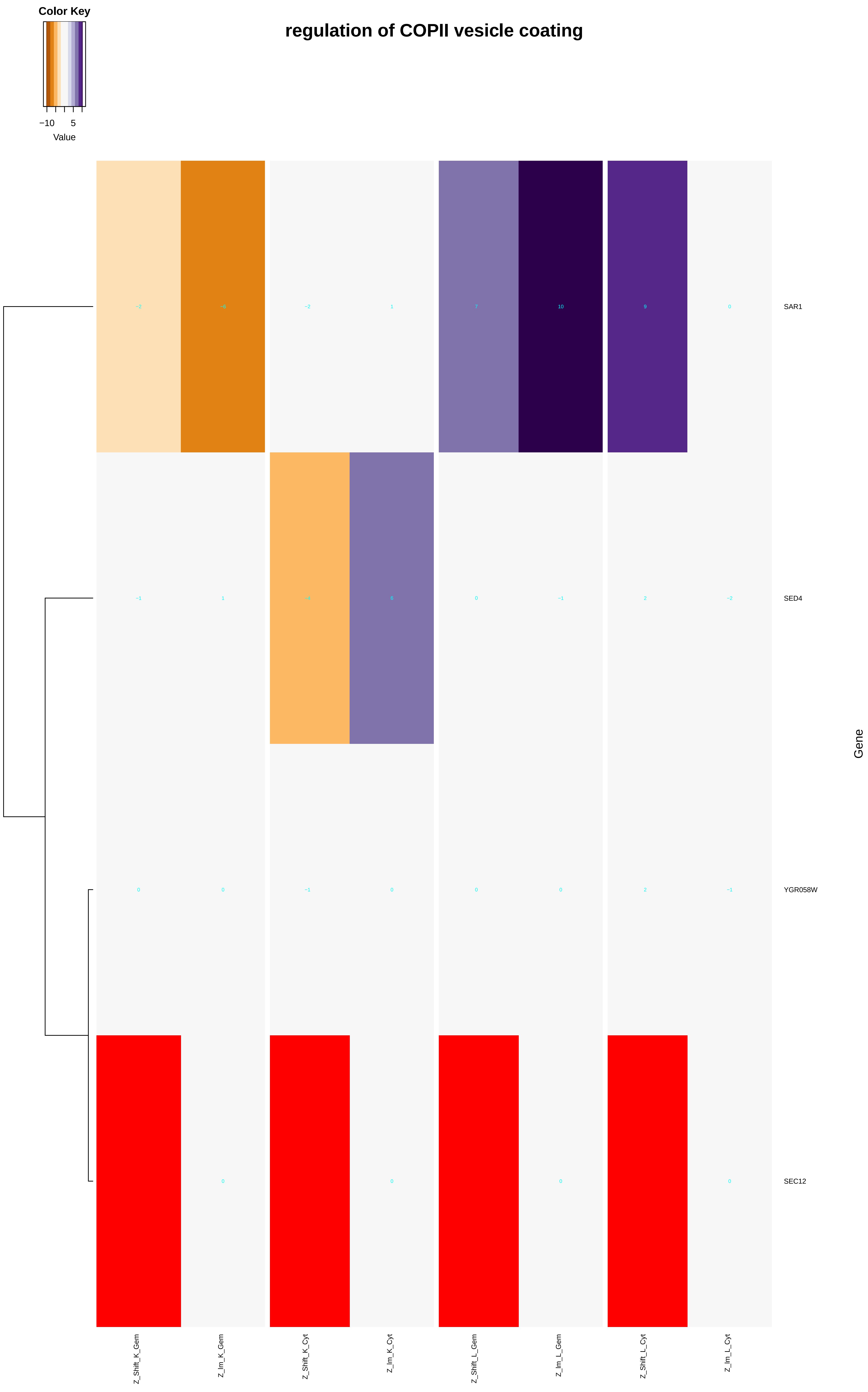

Color Key
−10
5
Value
regulation of COPII vesicle coating
SAR1
SED4
YGR058W
SEC12
Gene
Z_Shift_K_Gem
Z_Im_K_Gem
Z_Shift_K_Cyt
Z_Im_K_Cyt
Z_Shift_L_Gem
Z_Im_L_Gem
Z_Shift_L_Cyt
Z_Im_L_Cyt

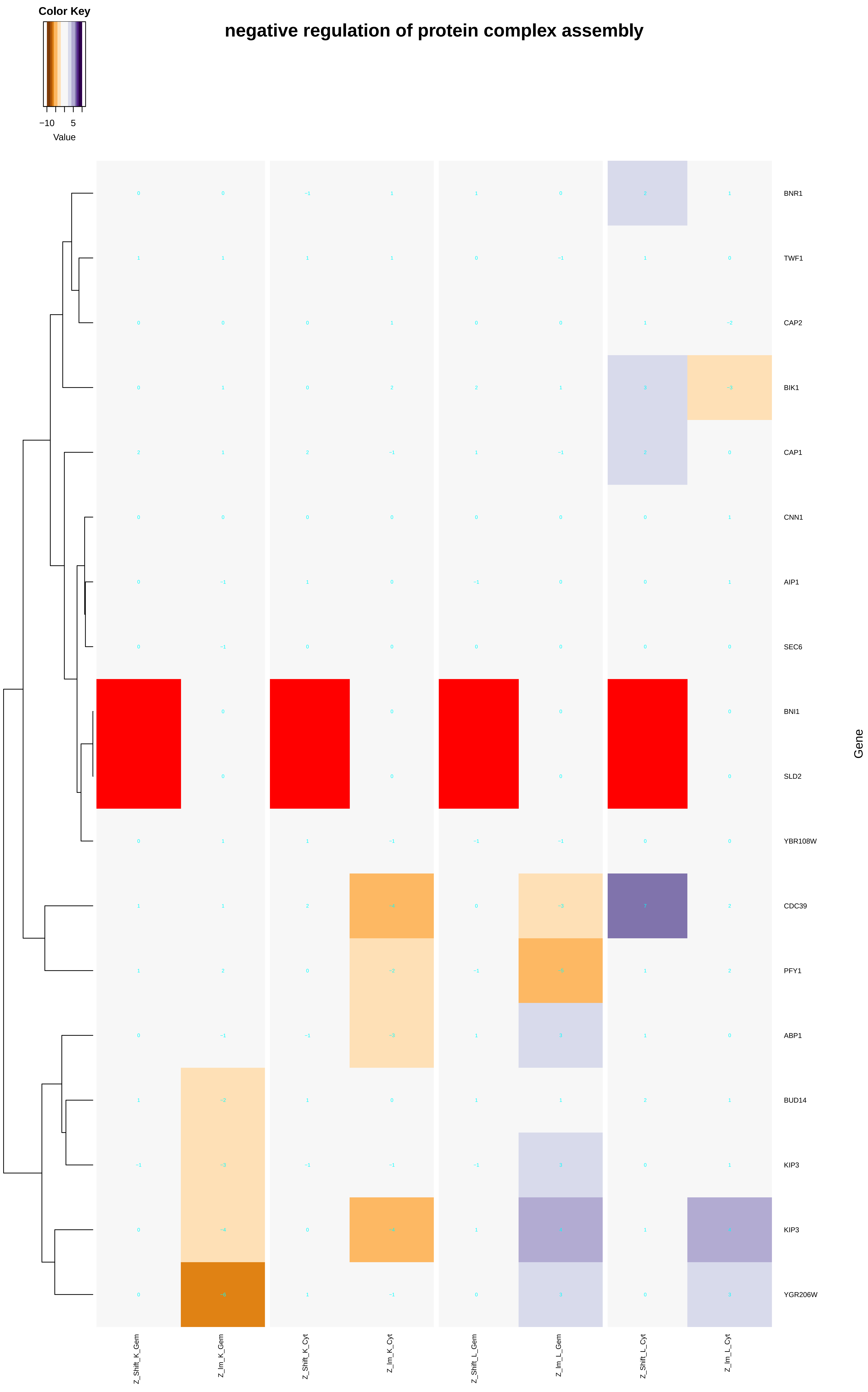

Color Key
−10
5
Value
negative regulation of protein complex assembly
BNR1
TWF1
CAP2
BIK1
CAP1
CNN1
AIP1
SEC6
BNI1
SLD2
YBR108W
CDC39
PFY1
ABP1
BUD14
KIP3
KIP3
YGR206W
Gene
Z_Shift_K_Gem
Z_lm_K_Gem
Z_Shift_K_Cyt
Z_lm_K_Cyt
Z_Shift_L_Gem
Z_lm_L_Gem
Z_Shift_L_Cyt
Z_lm_L_Cyt

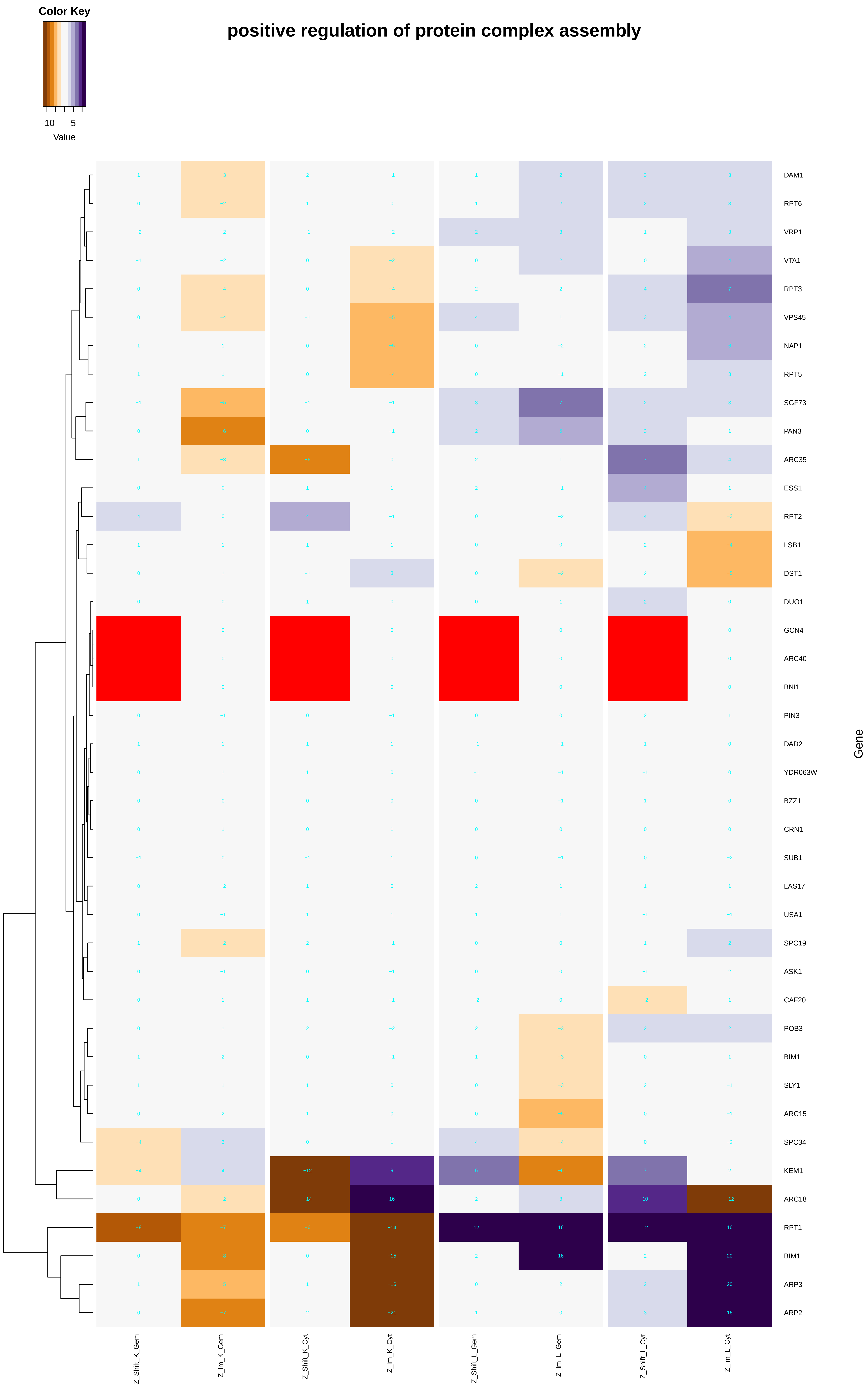

Color Key
−10
5
Value
positive regulation of protein complex assembly
DAM1
RPT6
VRP1
VTA1
RPT3
VPS45
NAP1
RPT5
SGF73
PAN3
ARC35
ESS1
RPT2
LSB1
DST1
DUO1
GCN4
ARC40
BNI1
PIN3
DAD2
YDR063W
BZZ1
CRN1
SUB1
LAS17
USA1
SPC19
ASK1
CAF20
POB3
BIM1
SLY1
ARC15
SPC34
KEM1
ARC18
RPT1
BIM1
ARP3
ARP2
Gene
Z_Shift_K_Gem
Z_lm_K_Gem
Z_Shift_K_Cyt
Z_lm_K_Cyt
Z_Shift_L_Gem
Z_lm_L_Gem
Z_Shift_L_Cyt
Z_lm_L_Cyt

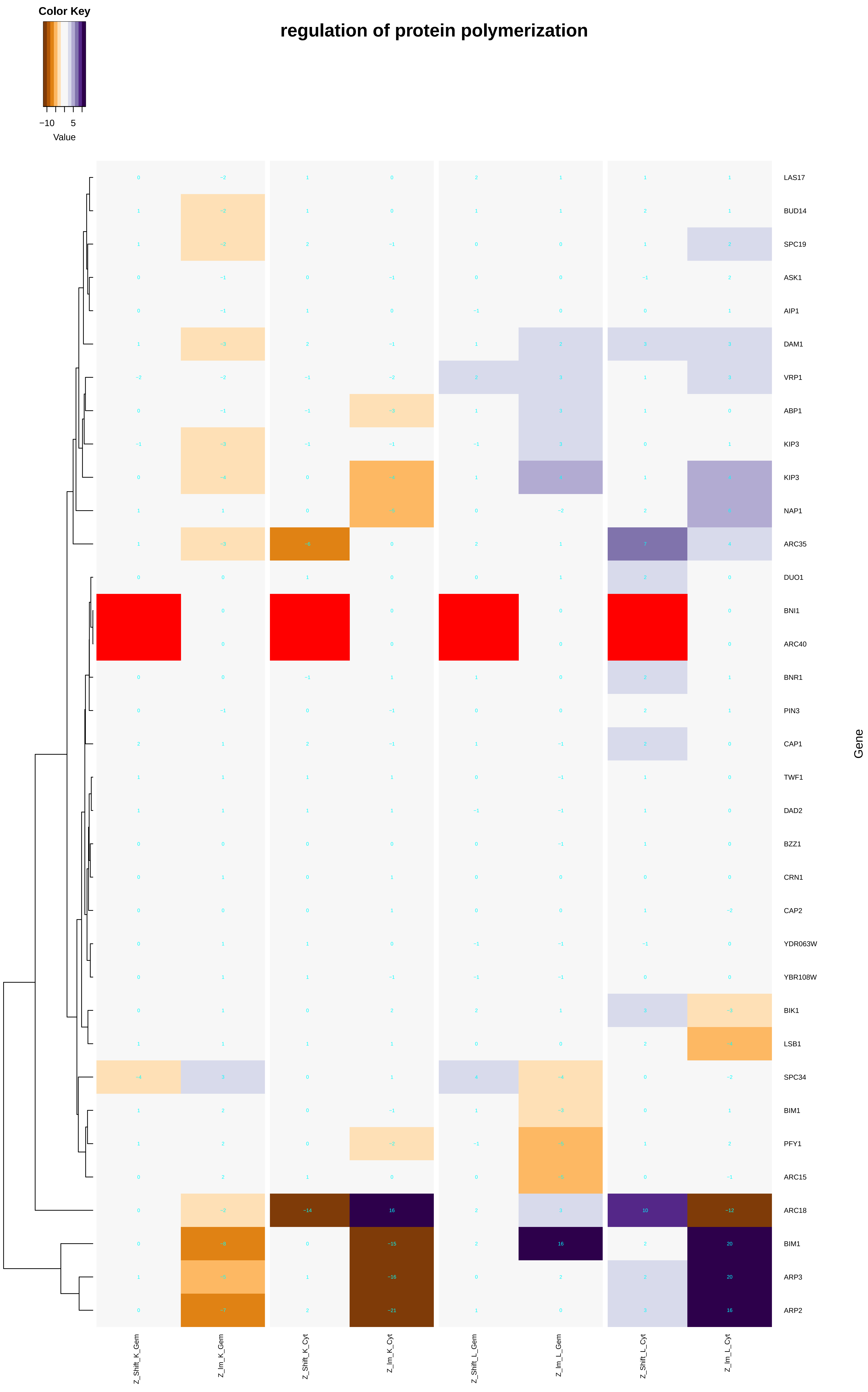

regulation of protein polymerization
Color Key
−10
5
Value
LAS17
BUD14
SPC19
ASK1
AIP1
DAM1
VRP1
ABP1
KIP3
KIP3
NAP1
ARC35
DUO1
BNI1
ARC40
BNR1
PIN3
CAP1
TWF1
DAD2
BZZ1
CRN1
CAP2
YDR063W
YBR108W
BIK1
LSB1
SPC34
BIM1
PFY1
ARC15
ARC18
BIM1
ARP3
ARP2
Gene
Z_Shift_K_Gem
Z_Im_K_Gem
Z_Shift_K_Cyt
Z_Im_K_Cyt
Z_Shift_L_Gem
Z_Im_L_Gem
Z_Shift_L_Cyt
Z_Im_L_Cyt

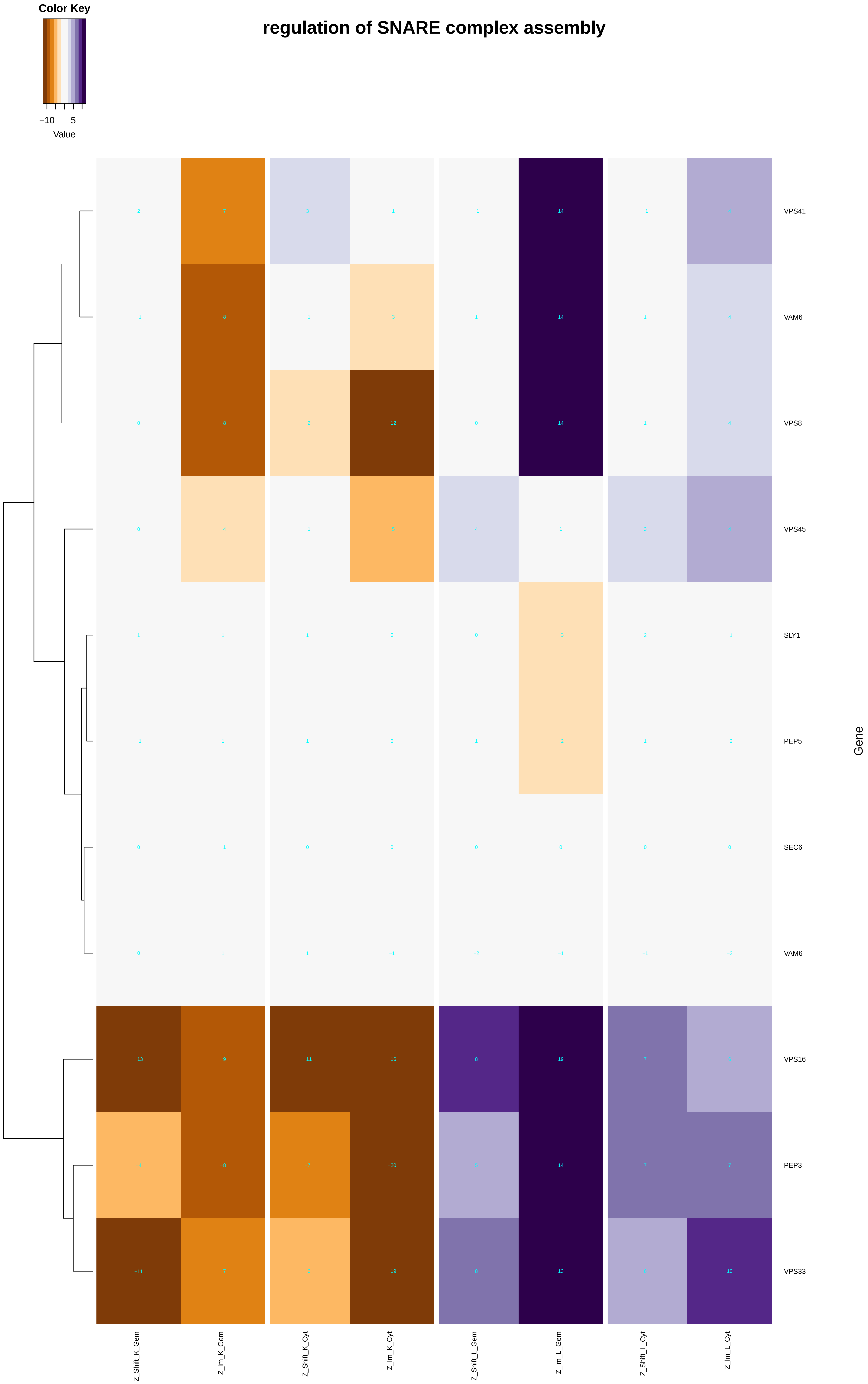

Color Key
−10
5
Value
regulation of SNARE complex assembly
VPS41
VAM6
VPS8
VPS45
SLY1
PEP5
SEC6
VAM6
VPS16
PEP3
VPS33
Gene
Z_Shift_K_Gem
Z_Im_K_Gem
Z_Shift_K_Cyt
Z_Im_K_Cyt
Z_Shift_L_Gem
Z_Im_L_Gem
Z_Shift_L_Cyt
Z_Im_L_Cyt

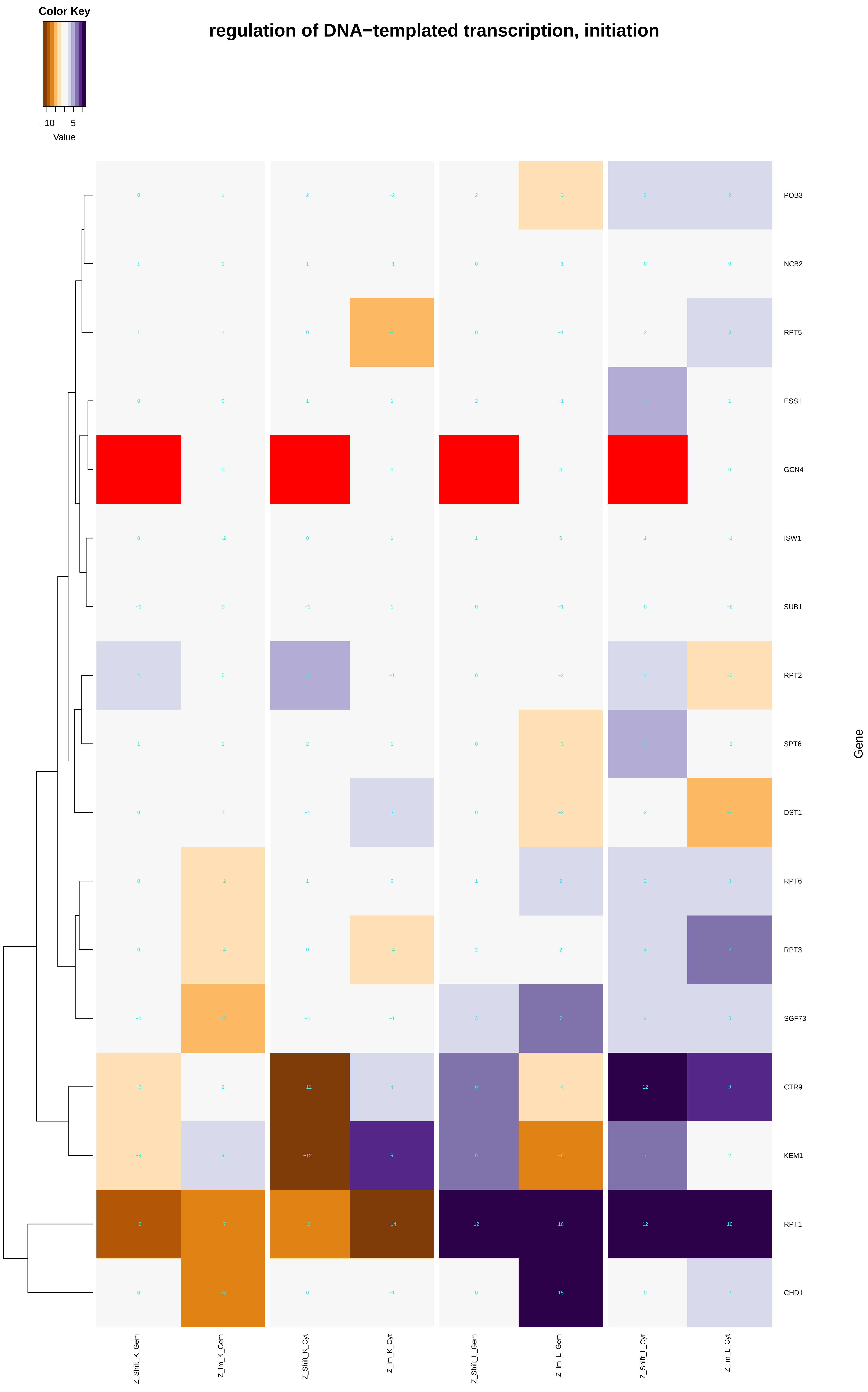

Color Key
−10
5
Value
regulation of DNA−templated transcription, initiation
POB3
NCB2
RPT5
ESS1
GCN4
ISW1
SUB1
RPT2
SPT6
DST1
RPT6
RPT3
SGF73
CTR9
KEM1
RPT1
CHD1
Gene
Z_Shift_K_Gem
Z_Im_K_Gem
Z_Shift_K_Cyt
Z_Im_K_Cyt
Z_Shift_L_Gem
Z_Im_L_Gem
Z_Shift_L_Cyt
Z_Im_L_Cyt

negative regulation of protein polymerization

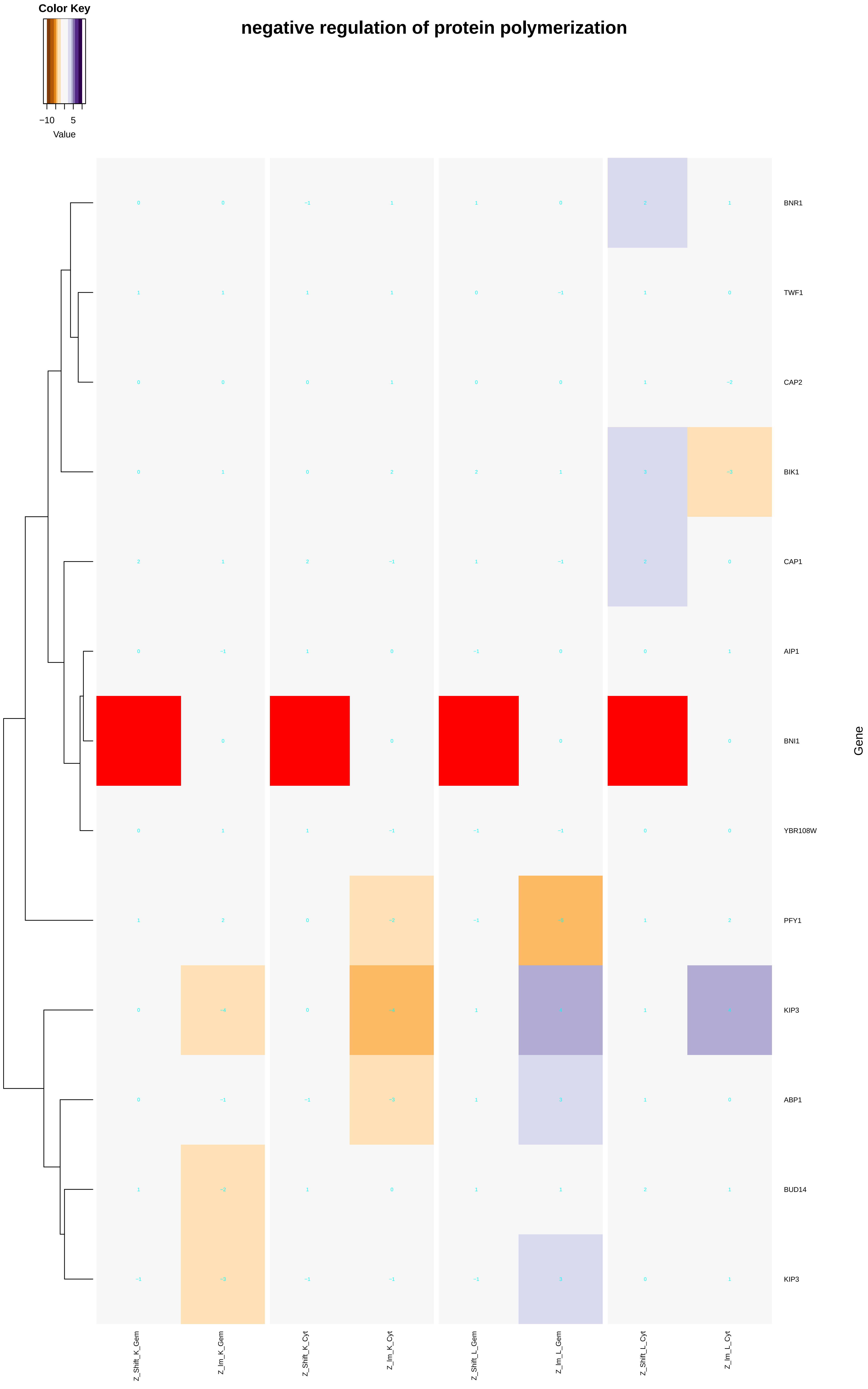

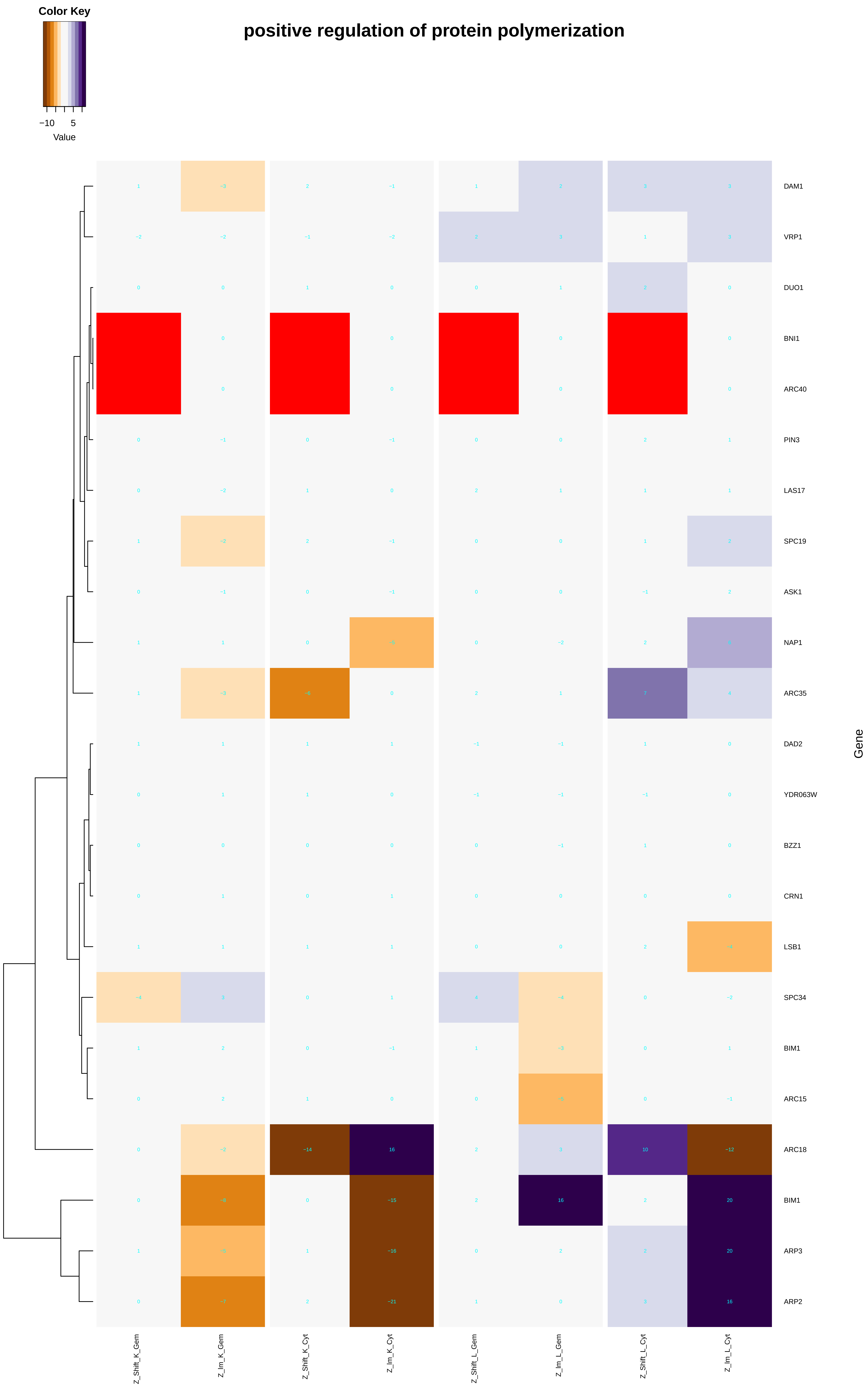
Color Key
−10
5
Value
positive regulation of protein polymerization
DAM1
VRP1
DUO1
BNI1
ARC40
PIN3
LAS17
SPC19
ASK1
NAP1
ARC35
DAD2
YDR063W
BZZ1
CRN1
LSB1
SPC34
BIM1
ARC15
ARC18
BIM1
ARP3
ARP2
Gene
Z_Shift_K_Gem
Z_Im_K_Gem
Z_Shift_K_Cyt
Z_Im_K_Cyt
Z_Shift_L_Gem
Z_Im_L_Gem
Z_Shift_L_Cyt
Z_Im_L_Cyt

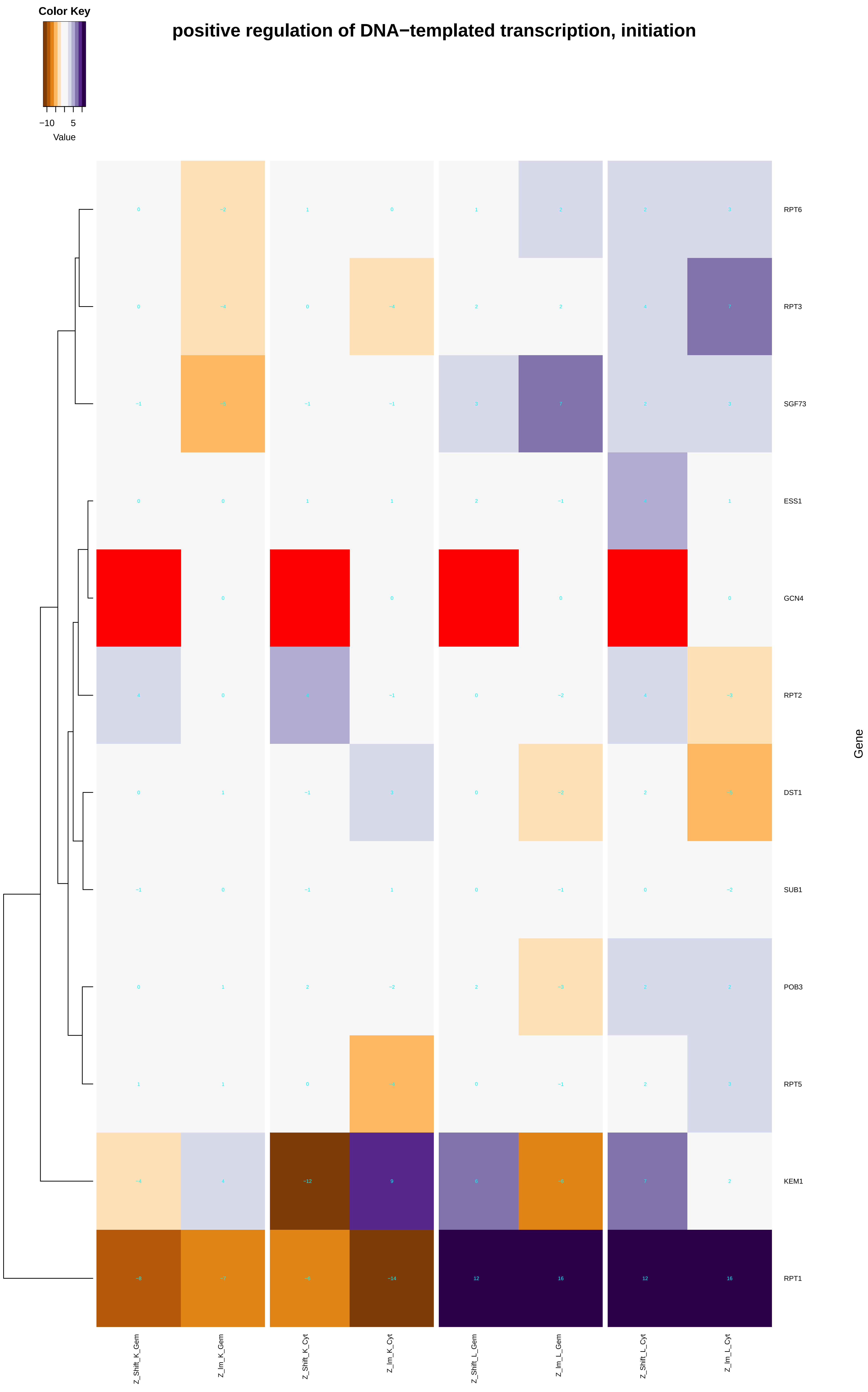

Color Key
−10
5
Value
positive regulation of DNA−templated transcription, initiation
RPT6
RPT3
SGF73
ESS1
GCN4
RPT2
DST1
SUB1
POB3
RPT5
KEM1
RPT1
Gene
Z_Shift_K_Gem
Z_Im_K_Gem
Z_Shift_K_Cyt
Z_Im_K_Cyt
Z_Shift_L_Gem
Z_Im_L_Gem
Z_Shift_L_Cyt
Z_Im_L_Cyt

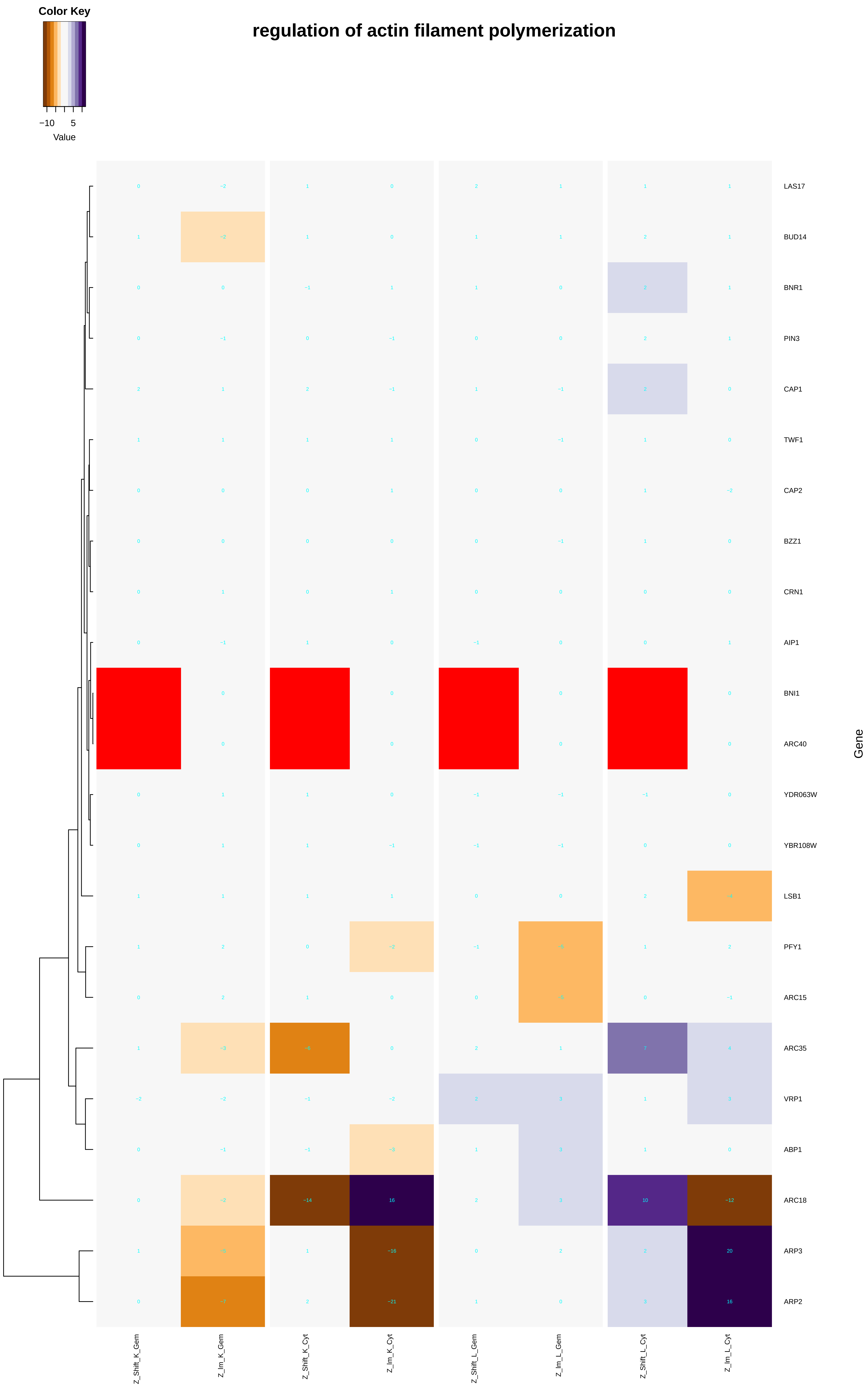

Color Key
−10
5
Value
regulation of actin filament polymerization
LAS17
BUD14
BNR1
PIN3
CAP1
TWF1
CAP2
BZZ1
CRN1
AIP1
BNI1
ARC40
YDR063W
YBR108W
LSB1
PFY1
ARC15
ARC35
VRP1
ABP1
ARC18
ARP3
ARP2
Gene
Z_Shift_K_Gem
Z_Im_K_Gem
Z_Shift_K_Cyt
Z_Im_K_Cyt
Z_Shift_L_Gem
Z_Im_L_Gem
Z_Shift_L_Cyt
Z_Im_L_Cyt

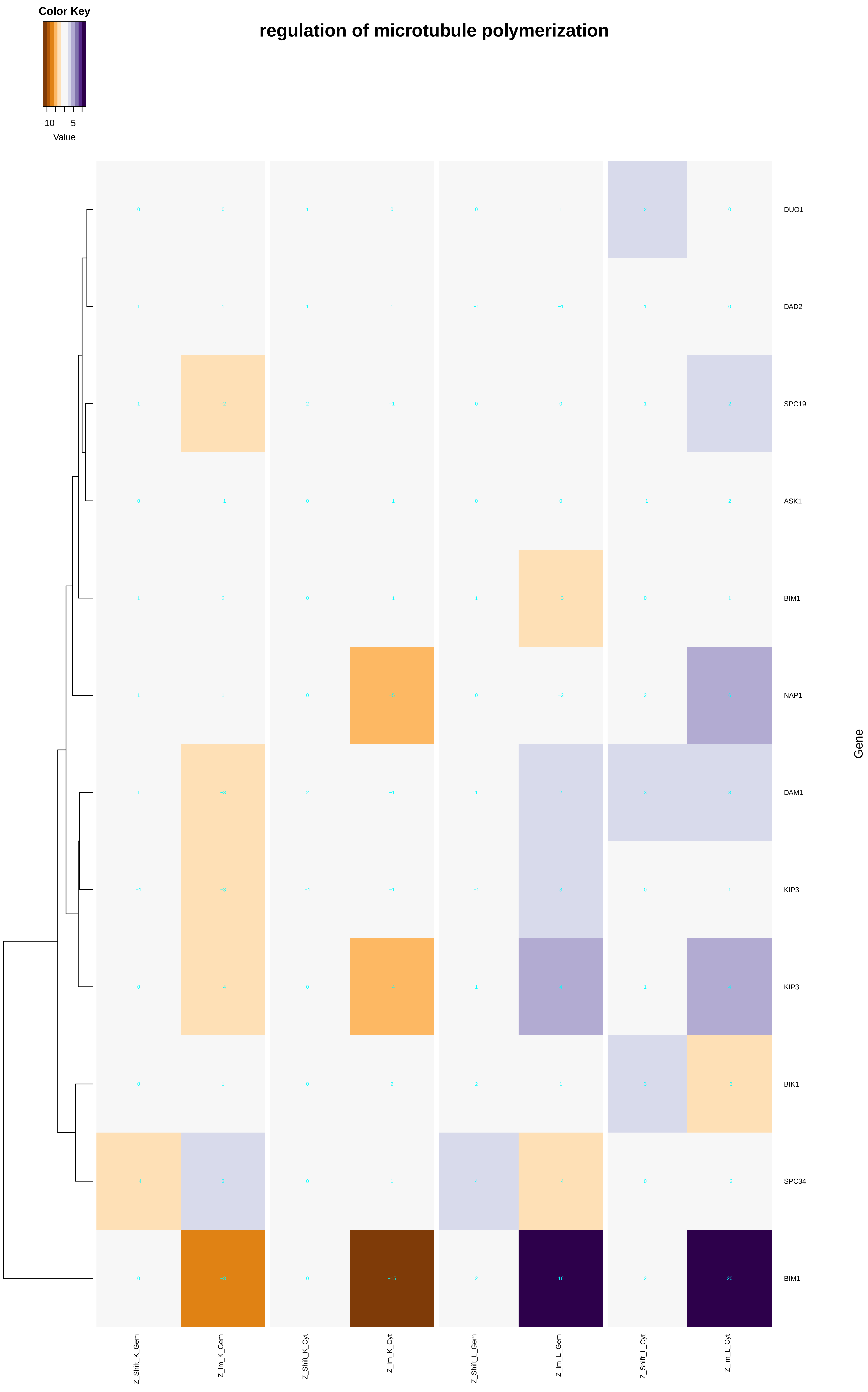

Color Key
−10
5
Value
regulation of microtubule polymerization
DUO1
DAD2
SPC19
ASK1
BIM1
NAP1
DAM1
KIP3
KIP3
BIK1
SPC34
BIM1
Gene
Z_Shift_K_Gem
Z_Im_K_Gem
Z_Shift_K_Cyt
Z_Im_K_Cyt
Z_Shift_L_Gem
Z_Im_L_Gem
Z_Shift_L_Cyt
Z_Im_L_Cyt

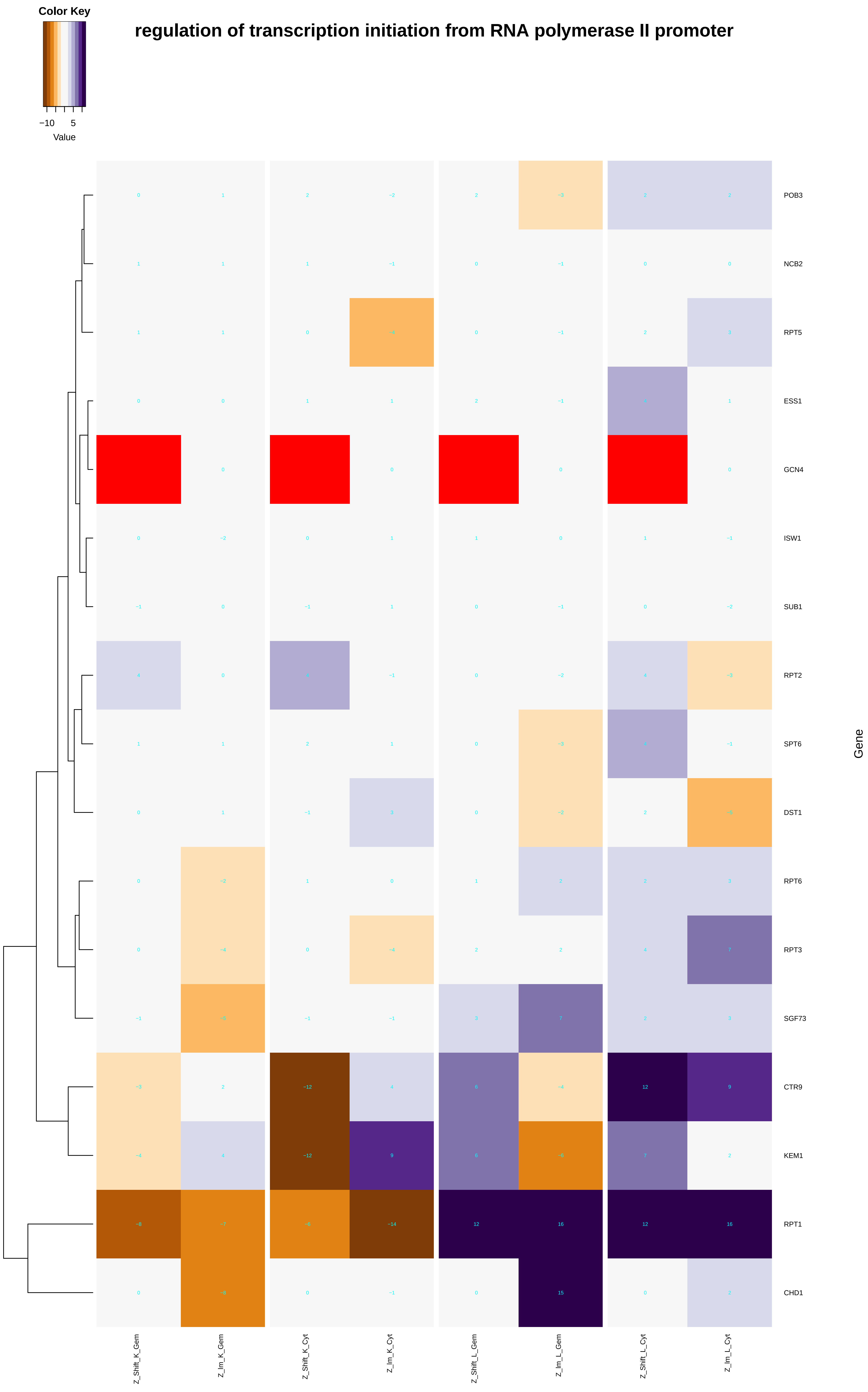
Color Key
−10
5
Value
regulation of transcription initiation from RNA polymerase II promoter
POB3
NCB2
RPT5
ESS1
GCN4
ISW1
SUB1
RPT2
SPT6
DST1
RPT6
RPT3
SGF73
CTR9
KEM1
RPT1
CHD1
Gene
Z_Shift_K_Gem
Z_lm_K_Gem
Z_Shift_K_Cyt
Z_lm_K_Cyt
Z_Shift_L_Gem
Z_lm_L_Gem
Z_Shift_L_Cyt
Z_lm_L_Cyt

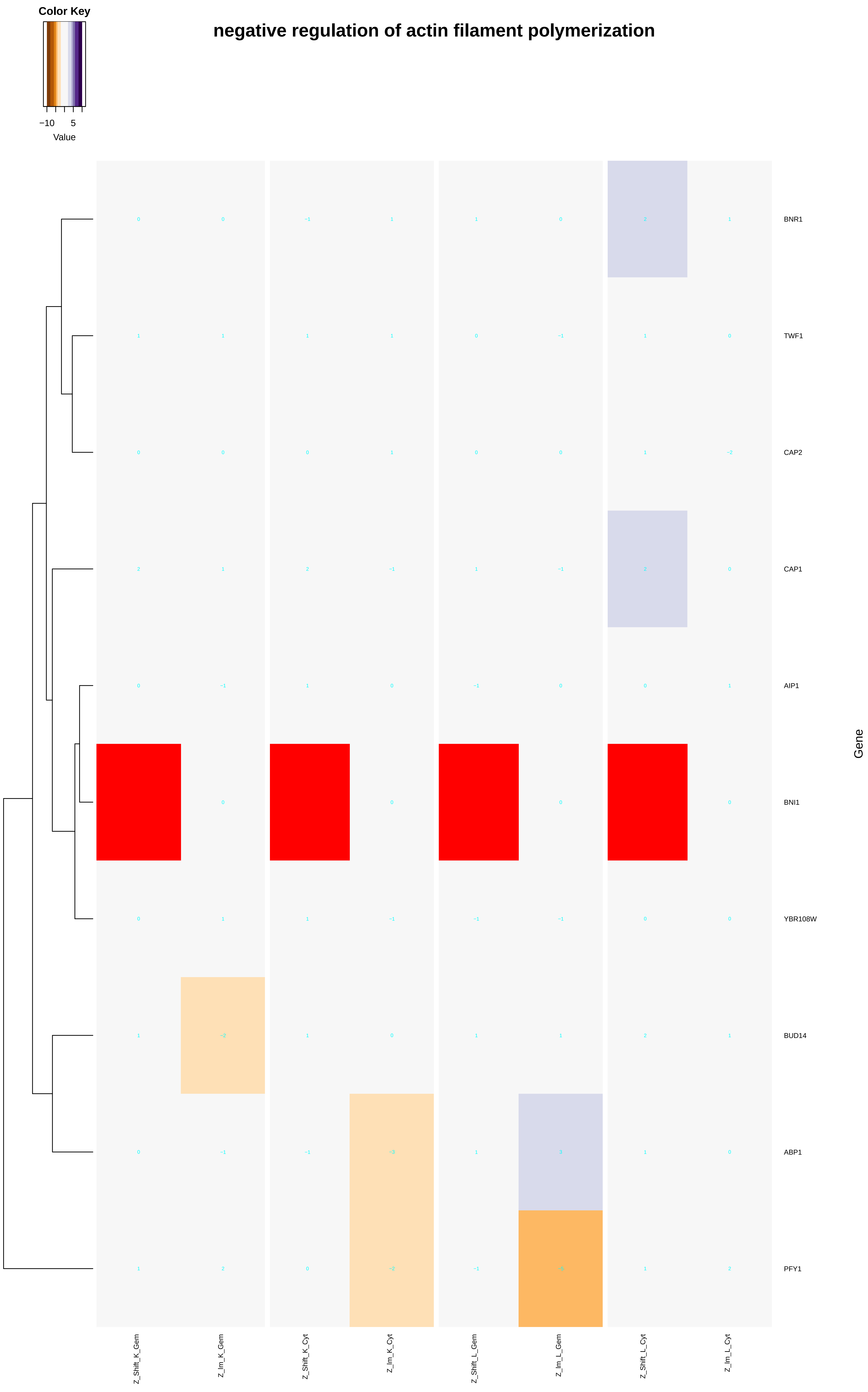

Color Key
−10
5
Value
negative regulation of actin filament polymerization
BNR1
TWF1
CAP2
CAP1
AIP1
BNI1
YBR108W
BUD14
ABP1
PFY1
Gene
Z_Shift_K_Gem
Z_Im_K_Gem
Z_Shift_K_Cyt
Z_Im_K_Cyt
Z_Shift_L_Gem
Z_Im_L_Gem
Z_Shift_L_Cyt
Z_Im_L_Cyt

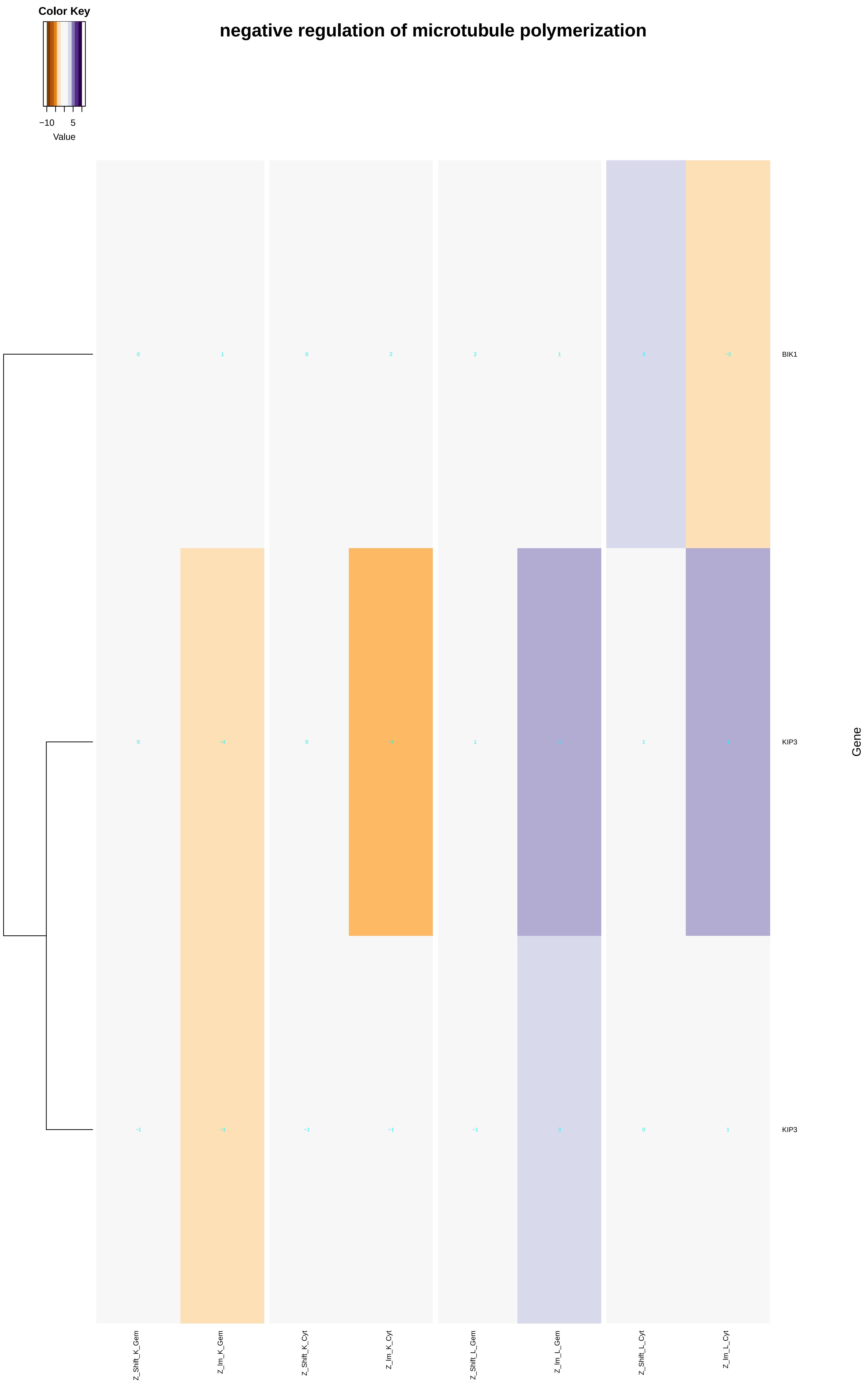
Color Key
negative regulation of microtubule polymerization
−10
5
Value
BIK1
KIP3
KIP3
Gene
Z_Shift_K_Gem
Z_Im_K_Gem
Z_Shift_K_Cyt
Z_Im_K_Cyt
Z_Shift_L_Gem
Z_Im_L_Gem
Z_Shift_L_Cyt
Z_Im_L_Cyt

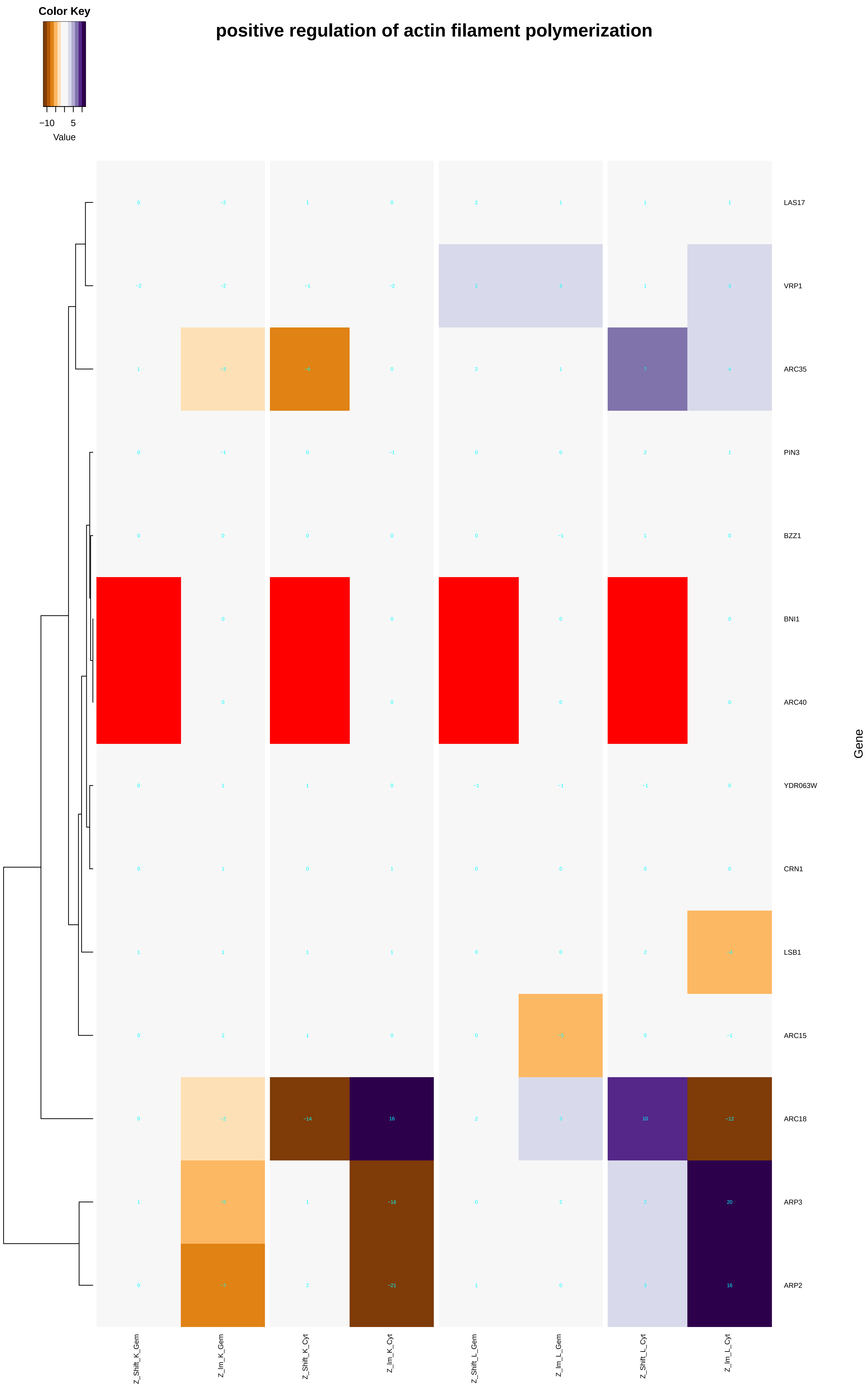

Color Key
−10
5
Value
positive regulation of actin filament polymerization
LAS17
VRP1
ARC35
PIN3
BZZ1
BNI1
ARC40
YDR063W
CRN1
LSB1
ARC15
ARC18
ARP3
ARP2
Gene
Z_Shift_K_Gem
Z_Im_K_Gem
Z_Shift_K_Cyt
Z_Im_K_Cyt
Z_Shift_L_Gem
Z_Im_L_Gem
Z_Shift_L_Cyt
Z_Im_L_Cyt

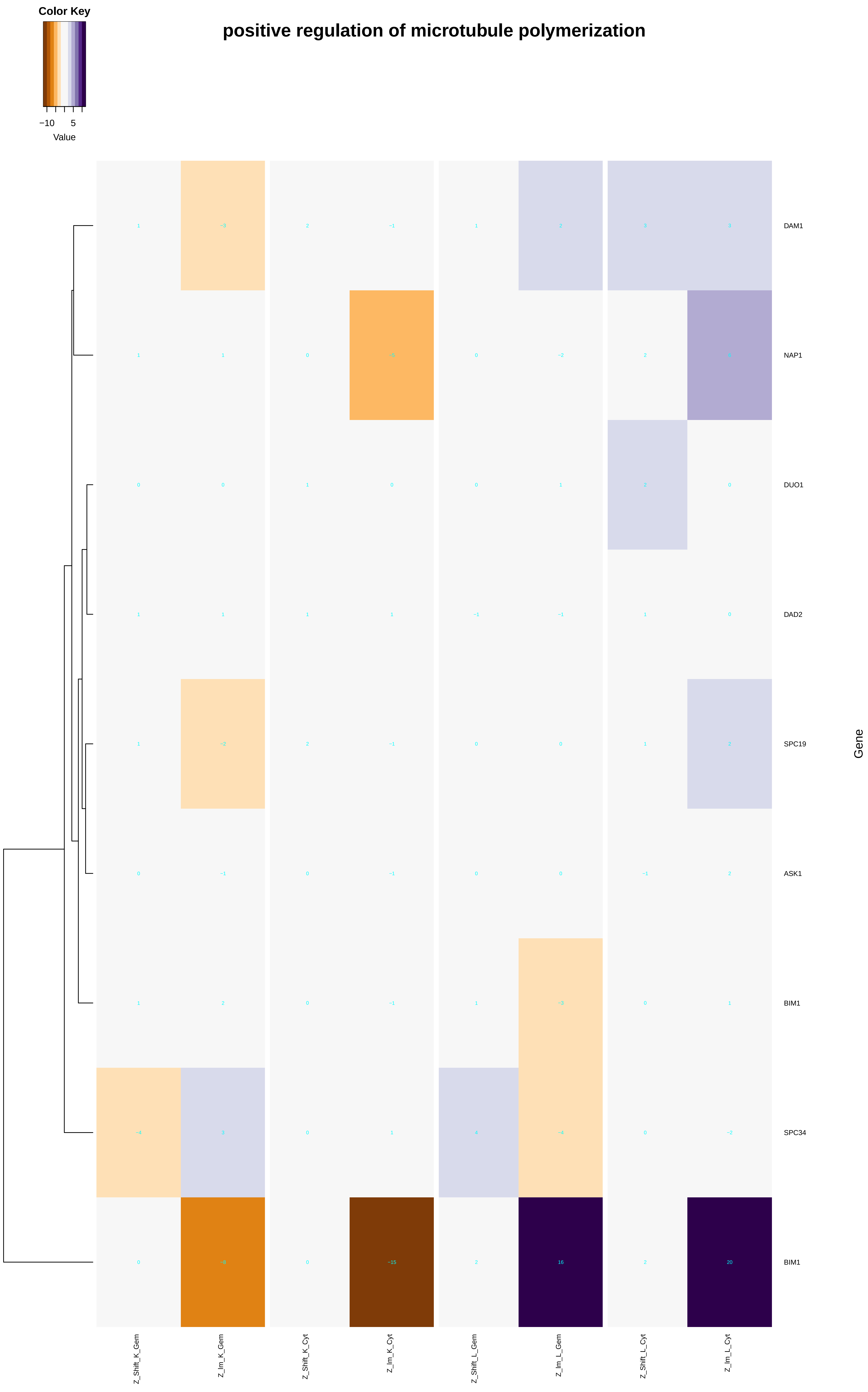
positive regulation of microtubule polymerization
Color Key
−10
5
Value
DAM1
NAP1
DUO1
DAD2
SPC19
ASK1
BIM1
SPC34
BIM1
Gene
Z_Shift_K_Gem
Z_Im_K_Gem
Z_Shift_K_Cyt
Z_Im_K_Cyt
Z_Shift_L_Gem
Z_Im_L_Gem
Z_Shift_L_Cyt
Z_Im_L_Cyt

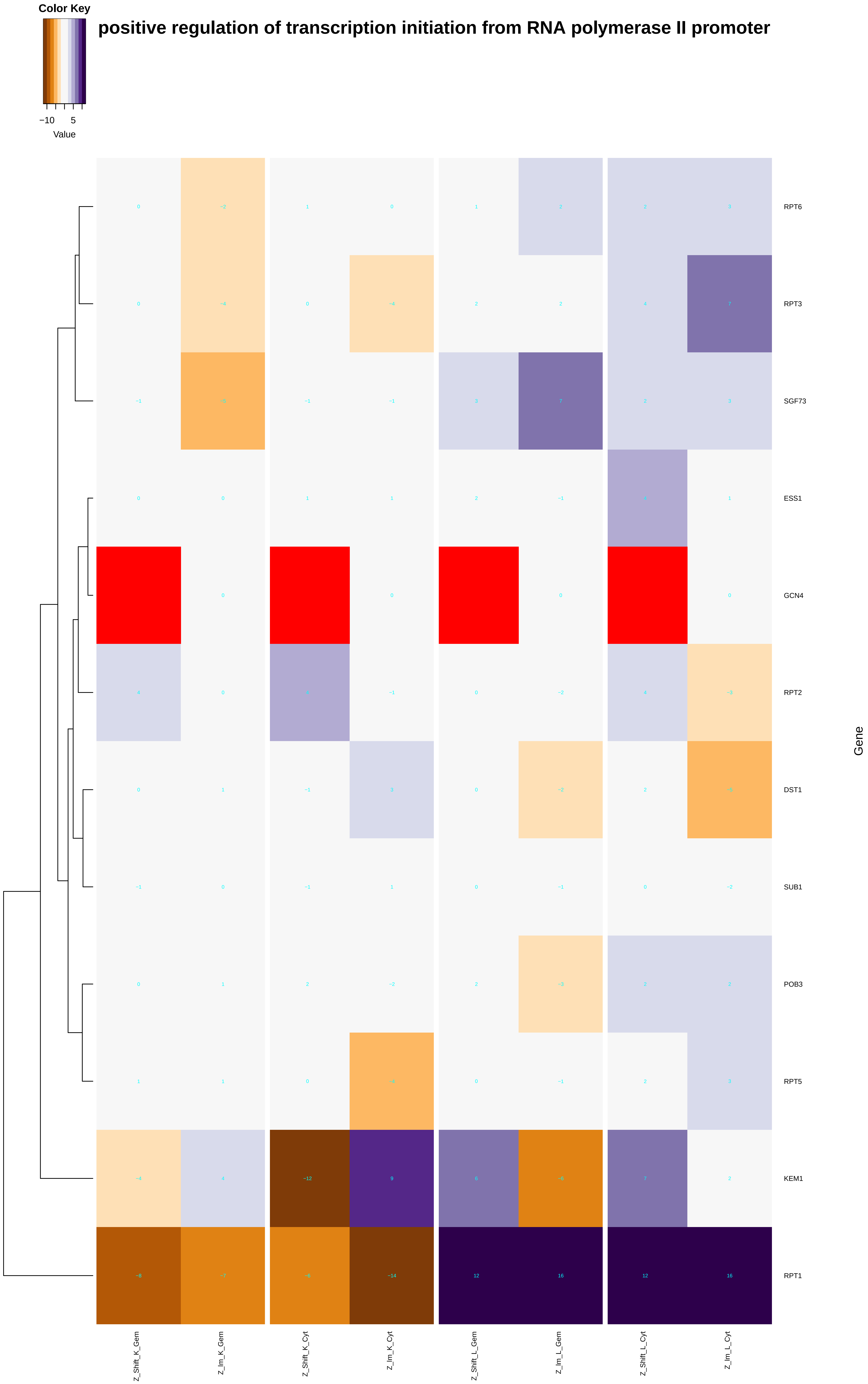
Color Key
−10
5
Value
positive regulation of transcription initiation from RNA polymerase II promoter
RPT6
RPT3
SGF73
ESS1
GCN4
RPT2
DST1
SUB1
POB3
RPT5
KEM1
RPT1
Gene
Z_Shift_K_Gem
Z_Im_K_Gem
Z_Shift_K_Cyt
Z_Im_K_Cyt
Z_Shift_L_Gem
Z_Im_L_Gem
Z_Shift_L_Cyt
Z_Im_L_Cyt

regulation of actin nucleation

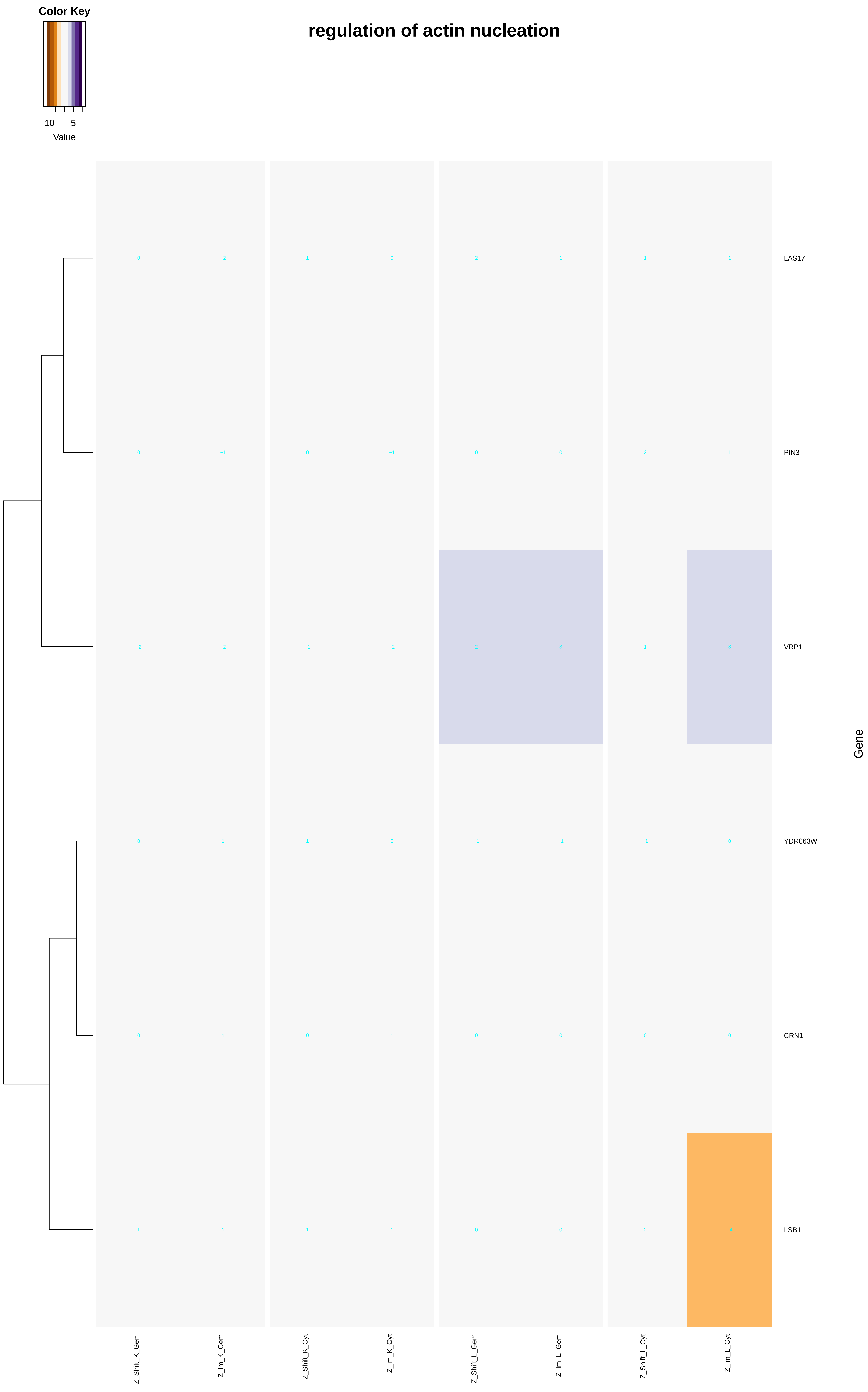

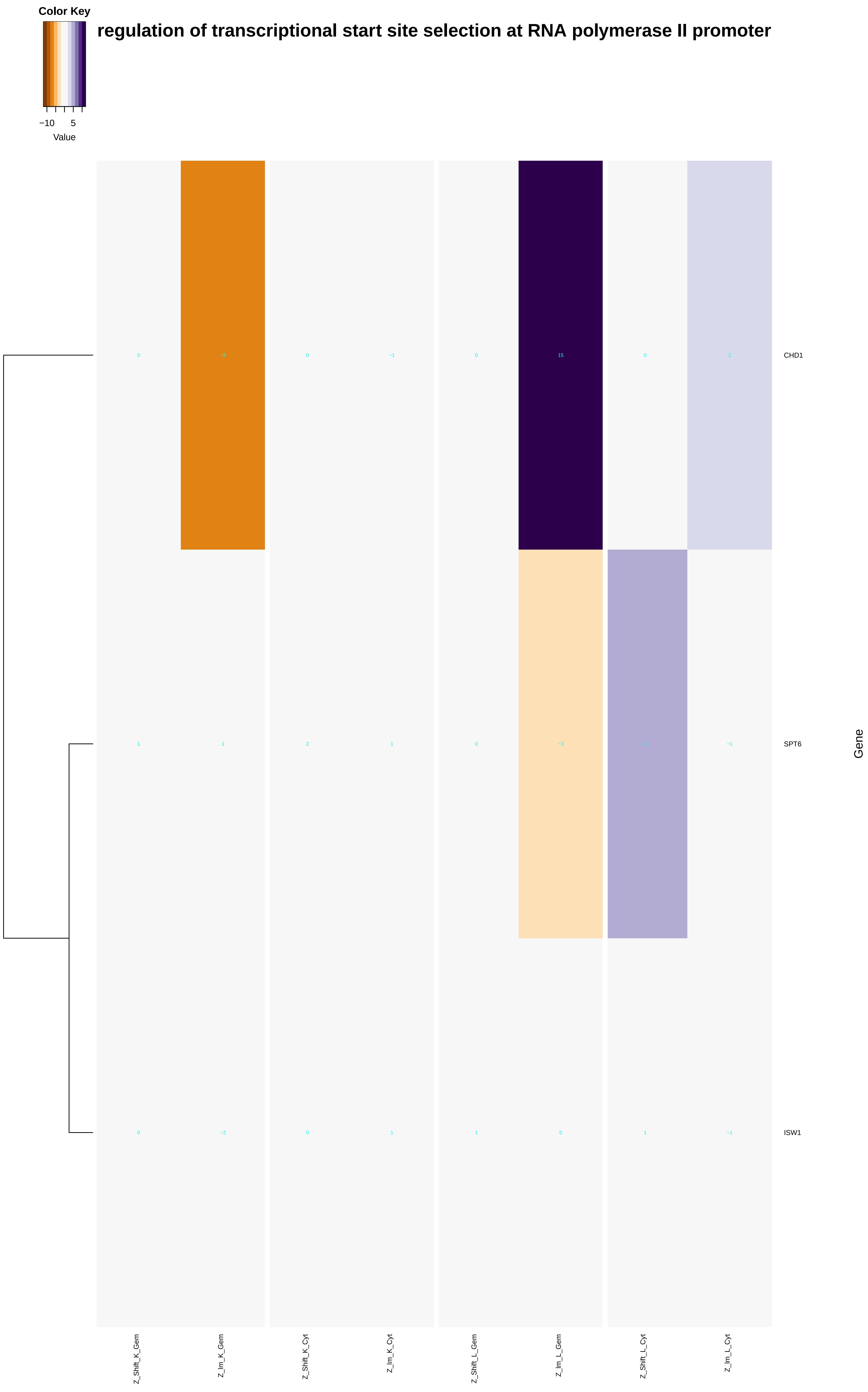

Color Key
−10
5
Value
regulation of transcriptional start site selection at RNA polymerase II promoter
CHD1
SPT6
ISW1
Gene
Z_Shift_K_Gem
Z_Im_K_Gem
Z_Shift_K_Cyt
Z_Im_K_Cyt
Z_Shift_L_Gem
Z_Im_L_Gem
Z_Shift_L_Cyt
Z_Im_L_Cyt
0
0
−1
0
15
0
2
1
1
2
1
0
−3
−1
0
−2
0
1
1
0
1
−1

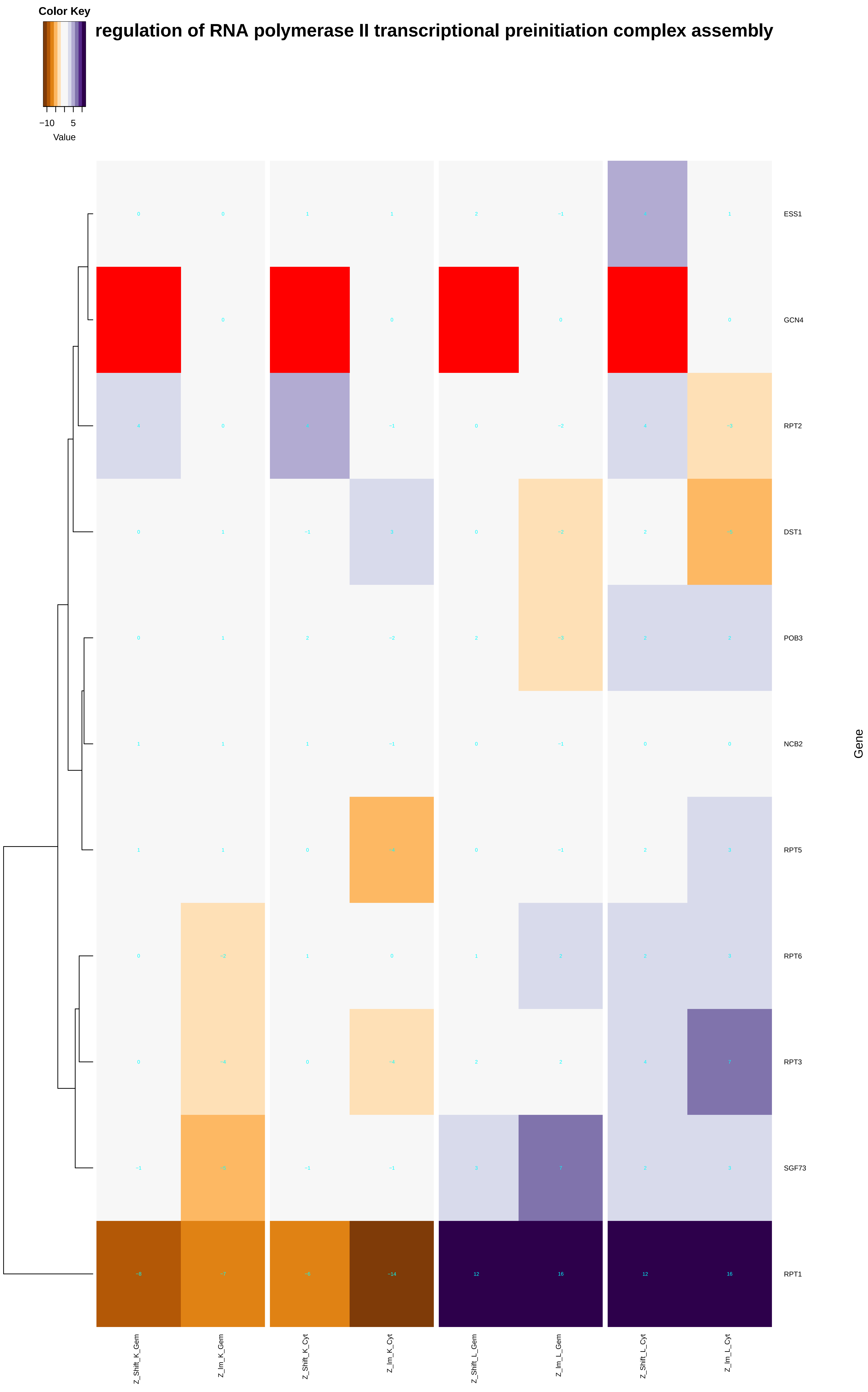
Color Key
−10
5
Value
regulation of RNA polymerase II transcriptional preinitiation complex assembly
ESS1
GCN4
RPT2
DST1
POB3
NCB2
RPT5
RPT6
RPT3
SGF73
RPT1
Gene
Z_Shift_K_Gem
Z_Im_K_Gem
Z_Shift_K_Cyt
Z_Im_K_Cyt
Z_Shift_L_Gem
Z_Im_L_Gem
Z_Shift_L_Cyt
Z_Im_L_Cyt

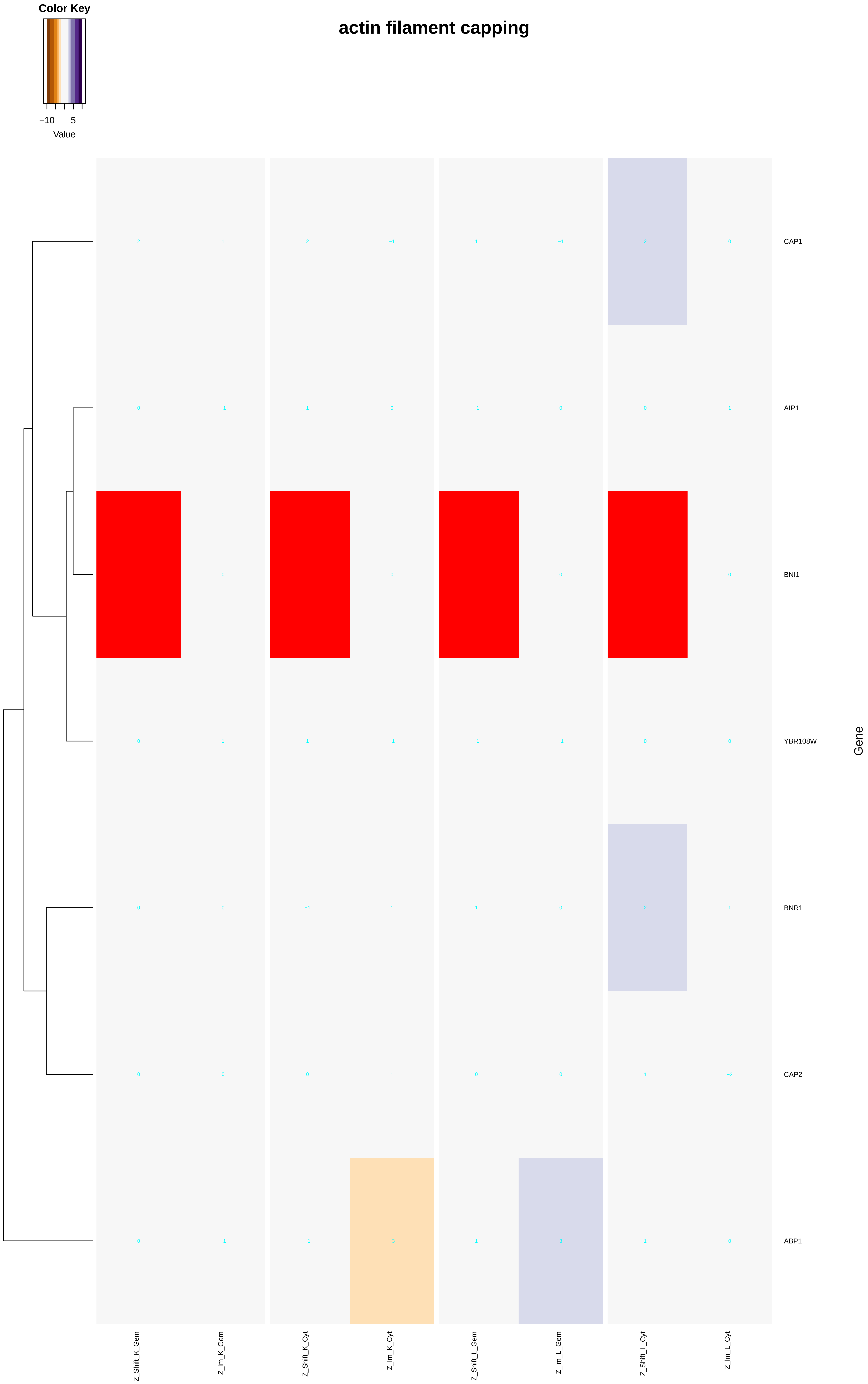

Color Key
−10
5
Value
actin filament capping
CAP1
AIP1
BNI1
YBR108W
BNR1
CAP2
ABP1
Gene
Z_Shift_K_Gem
Z_Im_K_Gem
Z_Shift_K_Cyt
Z_Im_K_Cyt
Z_Shift_L_Gem
Z_Im_L_Gem
Z_Shift_L_Cyt
Z_Im_L_Cyt

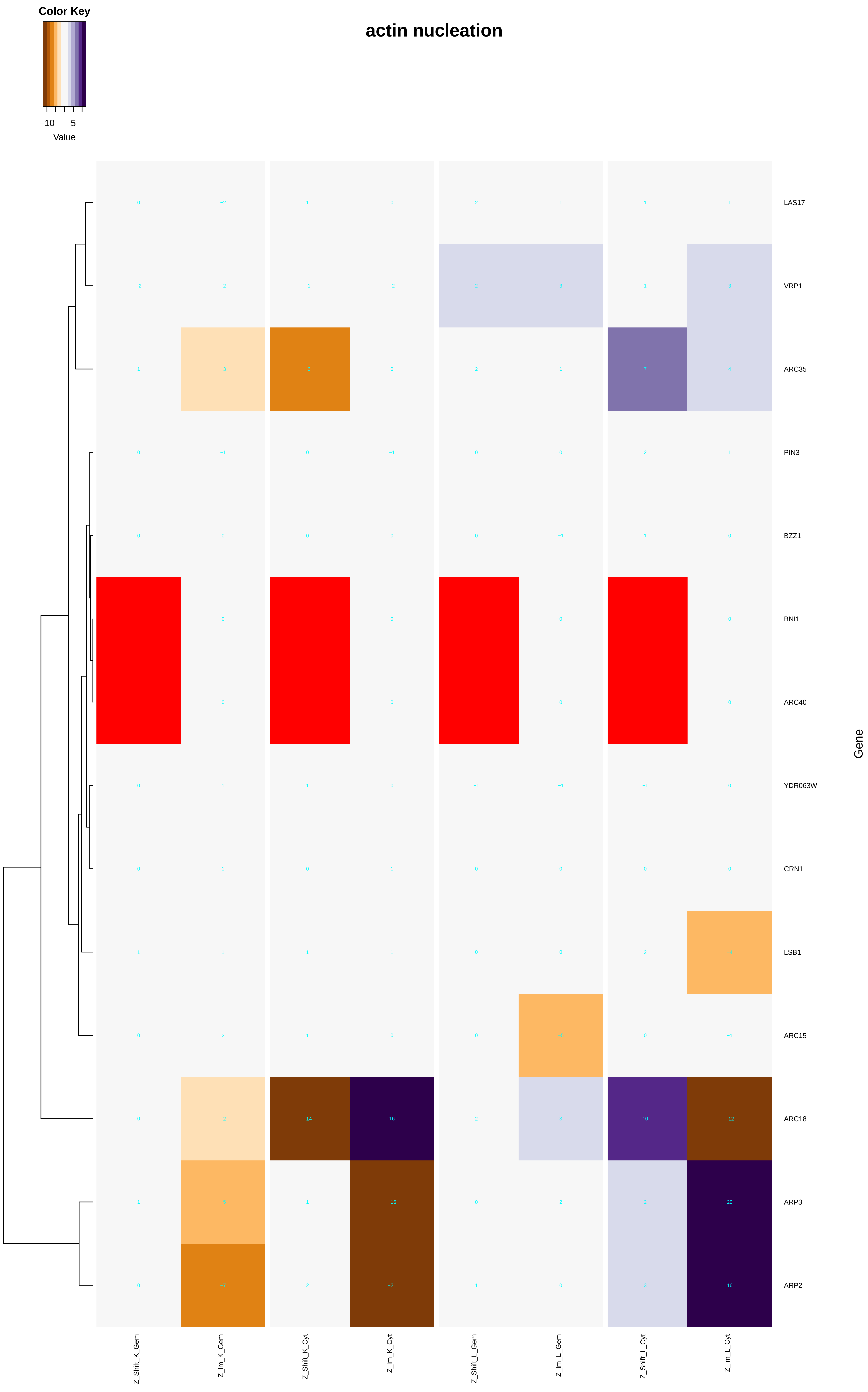

Color Key
−10 5
Value
actin nucleation
LAS17
VRP1
ARC35
PIN3
BZZ1
BNI1
ARC40
YDR063W
CRN1
LSB1
ARC15
ARC18
ARP3
ARP2
Gene
Z_Shift_K_Gem
Z_Im_K_Gem
Z_Shift_K_Cyt
Z_Im_K_Cyt
Z_Shift_L_Gem
Z_Im_L_Gem
Z_Shift_L_Cyt
Z_Im_L_Cyt

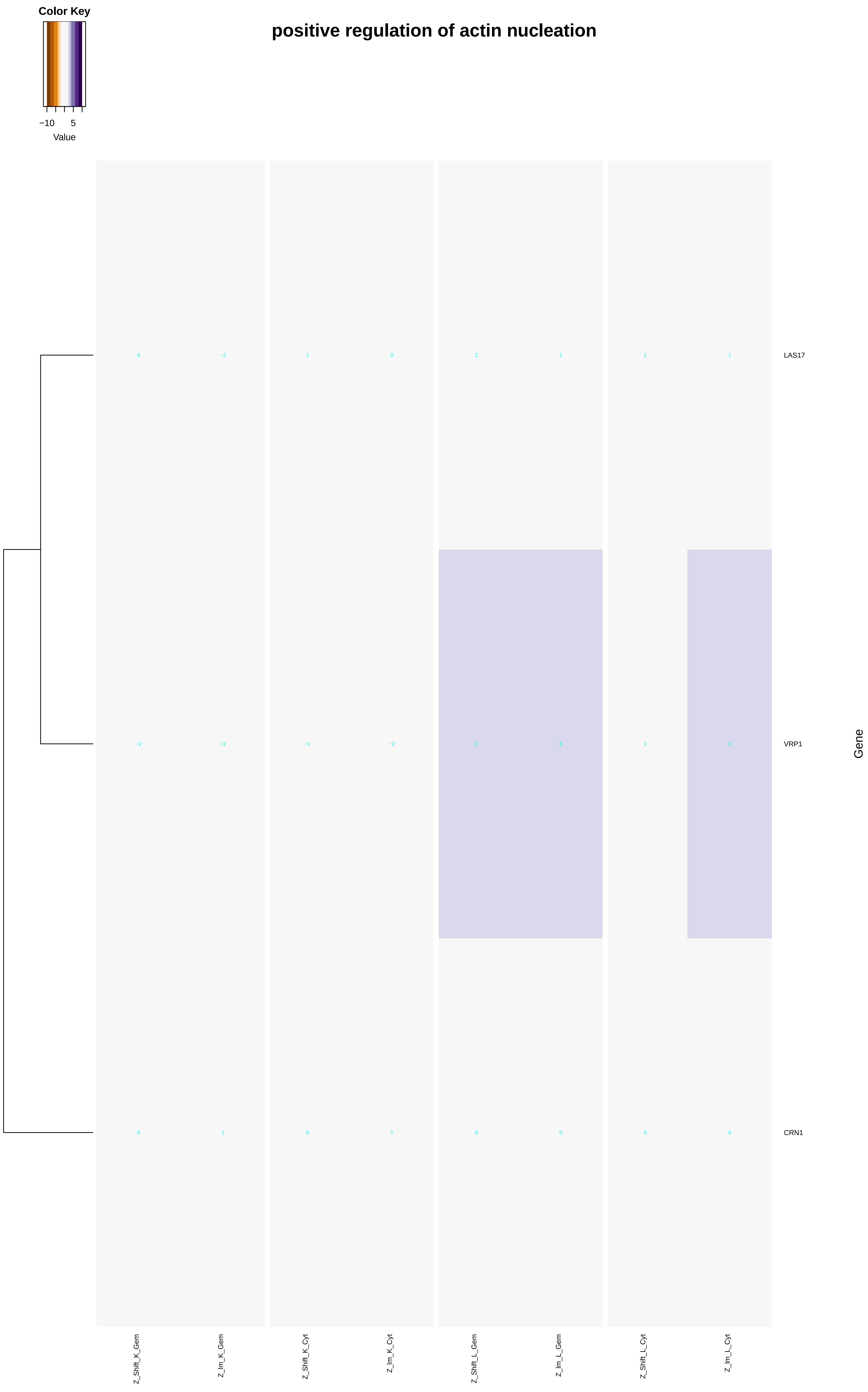
Color Key
−10 5
Value
positive regulation of actin nucleation
LAS17
VRP1
CRN1
Gene
0 −2 1 0 2 1 1 1
−2 −2 −1 −2 2 3 1 3
0 1 0 1 0 0 0 0
Z_Shift_K_Gem
Z_Im_K_Gem
Z_Shift_K_Cyt
Z_Im_K_Cyt
Z_Shift_L_Gem
Z_Im_L_Gem
Z_Shift_L_Cyt
Z_Im_L_Cyt

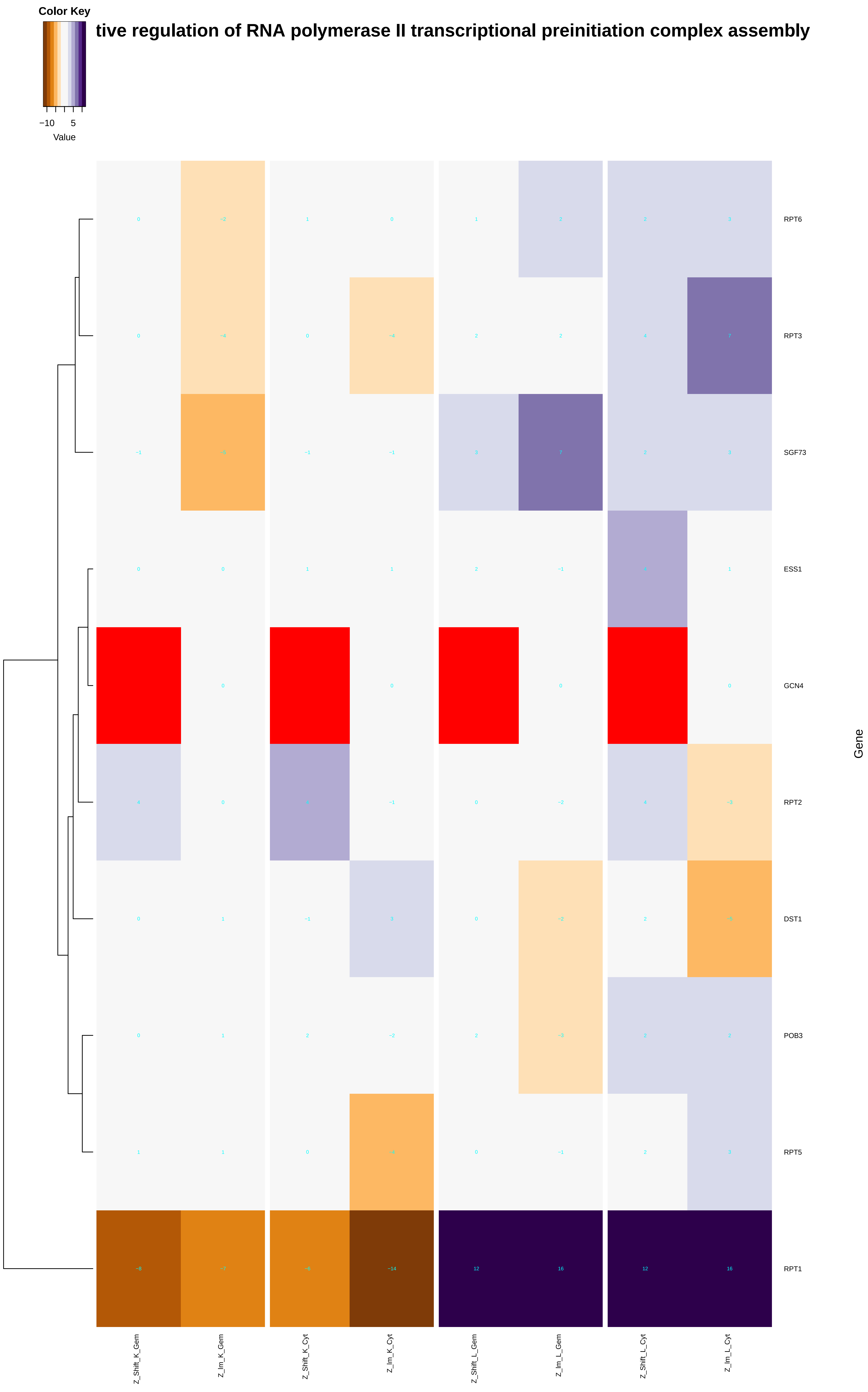
Color Key
−10 5
Value
tive regulation of RNA polymerase II transcriptional preinitiation complex assembly
RPT6
RPT3
SGF73
ESS1
GCN4
RPT2
DST1
POB3
RPT5
RPT1
Gene
Z_Shift_K_Gem
Z_Im_K_Gem
Z_Shift_K_Cyt
Z_Im_K_Cyt
Z_Shift_L_Gem
Z_Im_L_Gem
Z_Shift_L_Cyt
Z_Im_L_Cyt

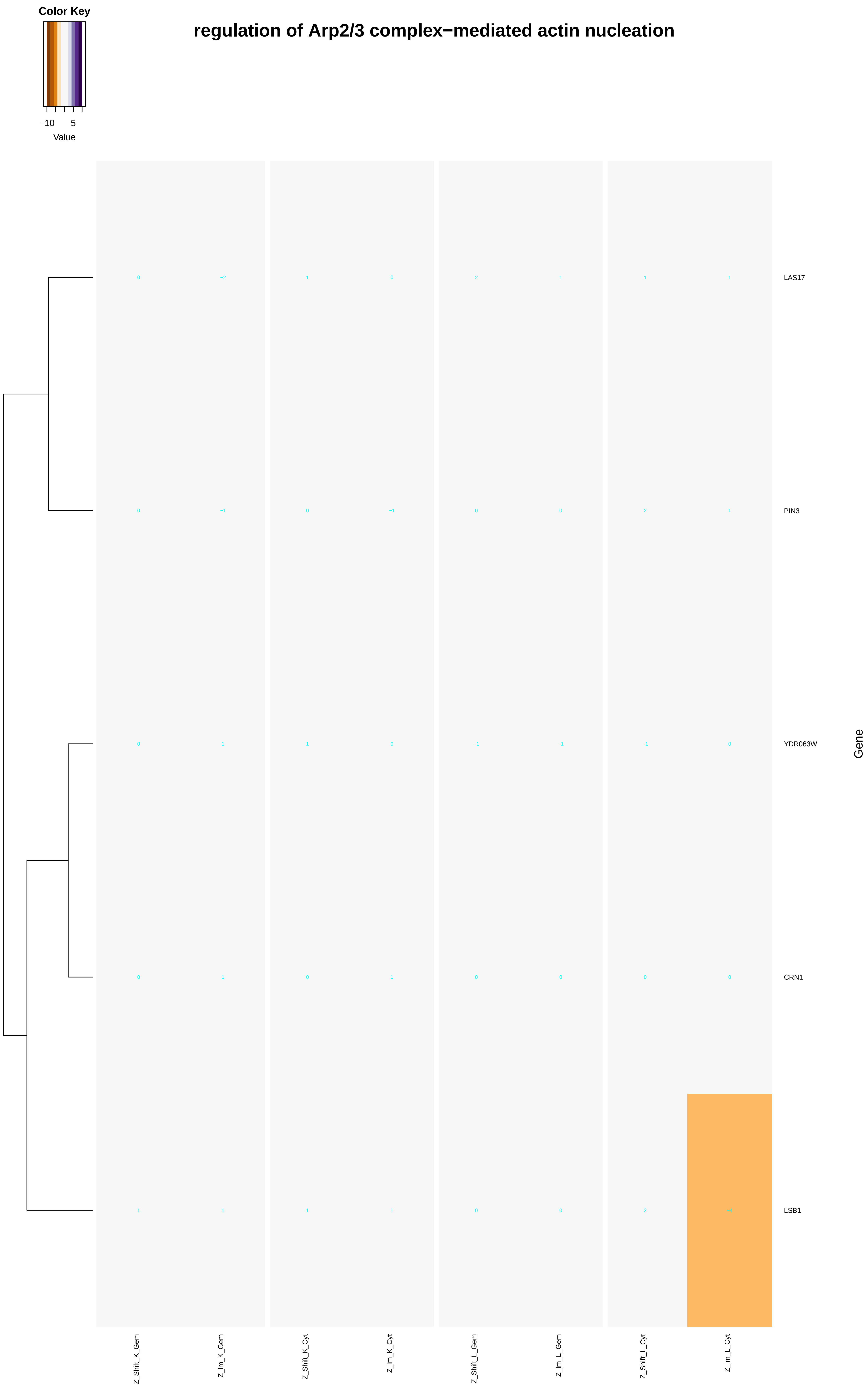

Color Key
−10
5
Value
regulation of Arp2/3 complex−mediated actin nucleation
LAS17
PIN3
YDR063W
CRN1
LSB1
Gene
Z_Shift_K_Gem
Z_Im_K_Gem
Z_Shift_K_Cyt
Z_Im_K_Cyt
Z_Shift_L_Gem
Z_Im_L_Gem
Z_Shift_L_Cyt
Z_Im_L_Cyt

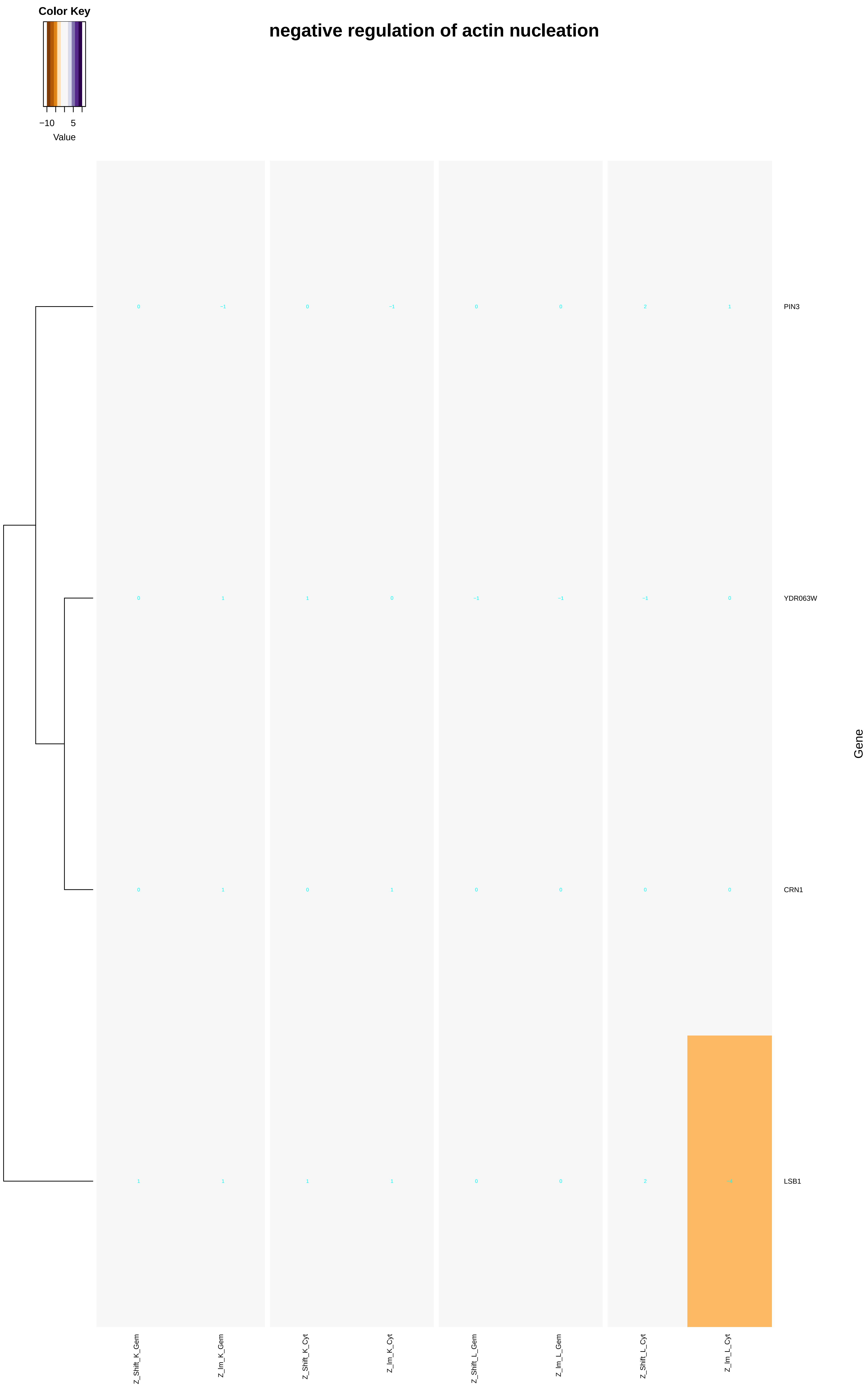
negative regulation of actin nucleation
Color Key
−10
5
Value
PIN3
YDR063W
CRN1
LSB1
Gene
Z_Shift_K_Gem
Z_Im_K_Gem
Z_Shift_K_Cyt
Z_Im_K_Cyt
Z_Shift_L_Gem
Z_Im_L_Gem
Z_Shift_L_Cyt
Z_Im_L_Cyt

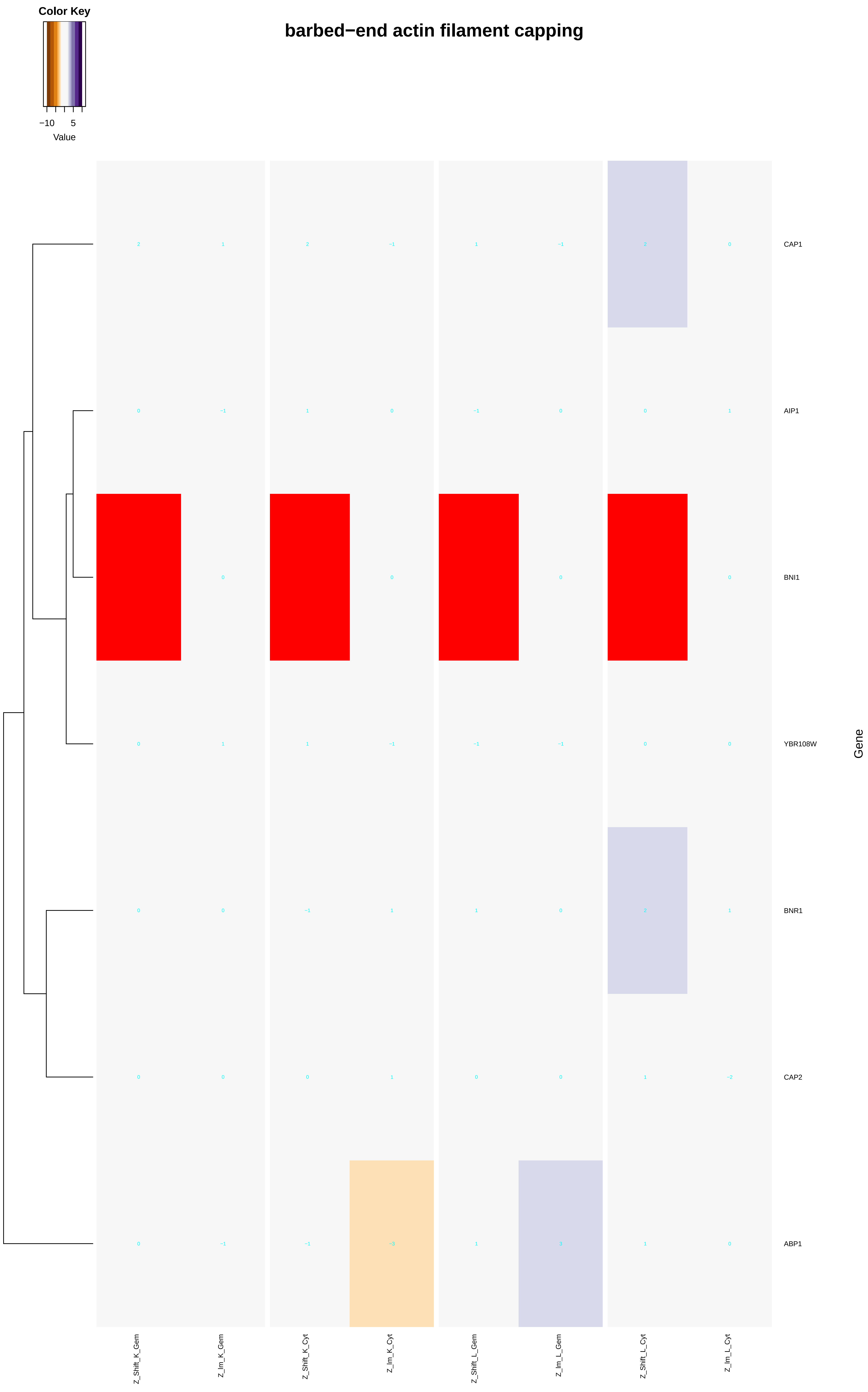

Color Key
−10
5
Value
barbed−end actin filament capping
CAP1
AIP1
BNI1
YBR108W
BNR1
CAP2
ABP1
Gene
Z_Shift_K_Gem
Z_Im_K_Gem
Z_Shift_K_Cyt
Z_Im_K_Cyt
Z_Shift_L_Gem
Z_Im_L_Gem
Z_Shift_L_Cyt
Z_Im_L_Cyt

Arp2/3 complex−mediated actin nucleation

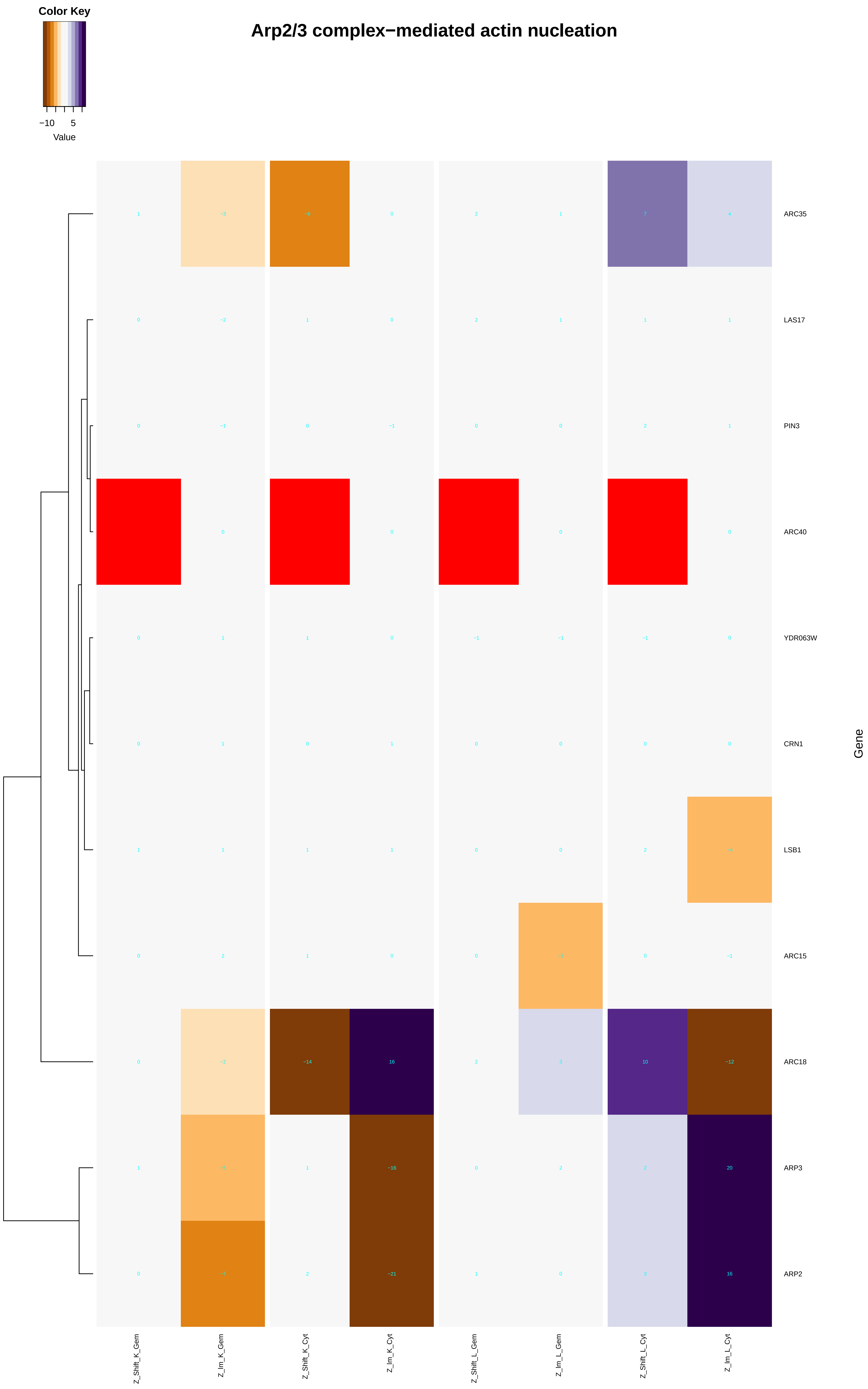

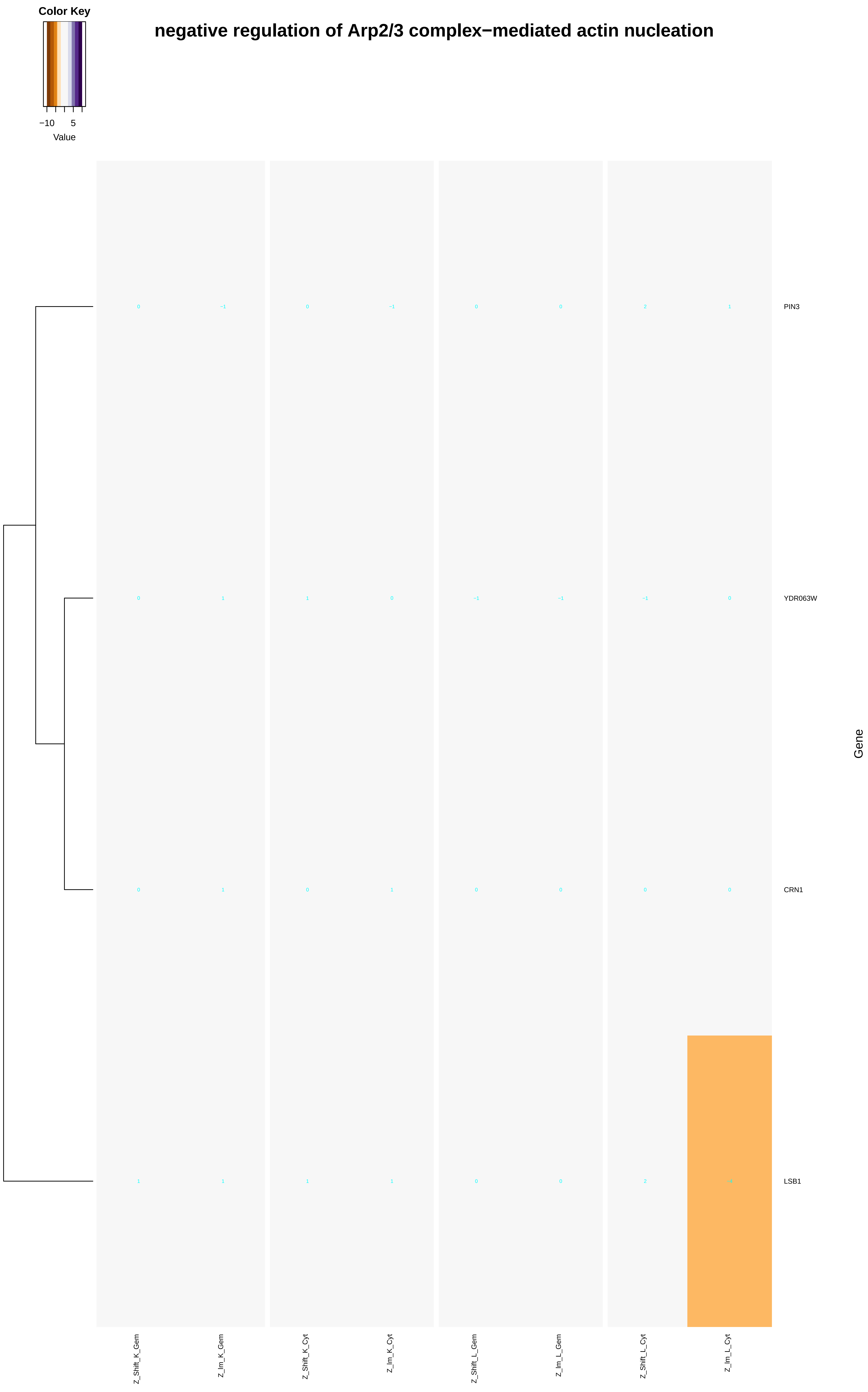

Color Key
−10
5
Value
negative regulation of Arp2/3 complex−mediated actin nucleation
PIN3
YDR063W
CRN1
LSB1
Gene
Z_Shift_K_Gem
Z_Im_K_Gem
Z_Shift_K_Cyt
Z_Im_K_Cyt
Z_Shift_L_Gem
Z_Im_L_Gem
Z_Shift_L_Cyt
Z_Im_L_Cyt
